# Neurotropic virus infections as the cause of immediate and delayed neuropathology

**DOI:** 10.1007/s00401-015-1511-3

**Published:** 2015-12-10

**Authors:** Martin Ludlow, Jeroen Kortekaas, Christiane Herden, Bernd Hoffmann, Dennis Tappe, Corinna Trebst, Diane E. Griffin, Hannah E. Brindle, Tom Solomon, Alan S. Brown, Debby van Riel, Katja C. Wolthers, Dasja Pajkrt, Peter Wohlsein, Byron E. E. Martina, Wolfgang Baumgärtner, Georges M. Verjans, Albert D. M. E. Osterhaus

**Affiliations:** Research Center for Emerging Infections and Zoonoses, University of Veterinary Medicine, Bünteweg 17, 30559 Hannover, Germany; Department of Virology, Central Veterinary Institute, Part of Wageningen University and Research Centre, Wageningen, The Netherlands; Institute of Veterinary Pathology, Justus-Liebig-University Gießen, Giessen, Germany; Institute of Diagnostic Virology, Friedrich-Loeffler-Institut, Greifswald-Insel Riems, Germany; Bernhard Nocht Institute for Tropical Medicine, Hamburg, Germany; German Centre for Infection Research (DZIF), Hamburg, Germany; Department of Neurology, Hannover Medical School, Hannover, Germany; W. Harry Feinstone Department of Molecular Microbiology and Immunology, Johns Hopkins Bloomberg School of Public Health, Baltimore, MD USA; Institute of Infection and Global Health, University of Liverpool, Liverpool, UK; Wellcome Trust Liverpool Glasgow Centre for Global Health Research, University of Liverpool, Liverpool, UK; NIHR Health Protection Research Unit in Emerging Infection and Zoonoses, Liverpool, UK; Department of Psychiatry, Columbia University Medical Center, New York State Psychiatric Institute, New York, NY USA; Department of Viroscience, Erasmus MC, Rotterdam, The Netherlands; Laboratory of Clinical Virology, Department of Medical Microbiology, Academic Medical Center, Amsterdam, The Netherlands; Department of Pediatric Infectious Diseases, Emma Children’s Hospital, Academic Medical Center, Amsterdam, The Netherlands; Department of Pathology, University of Veterinary Medicine, Hannover, Germany; Artemis One Health, Utrecht, The Netherlands; Center of Systems Neuroscience, Hannover, Germany

**Keywords:** Central nervous system, Neuropathology, Neuroinfectiology, Virus infection, Alphavirus, Bornavirus, Bunyavirus, Flavivirus, Herpesvirus, Influenza virus, Paramyxovirus, Picornavirus, Rhabdovirus

## Abstract

A wide range of viruses from different virus families in different geographical areas, may cause immediate or delayed neuropathological changes and neurological manifestations in humans and animals. Infection by neurotropic viruses as well as the resulting immune response can irreversibly disrupt the complex structural and functional architecture of the central nervous system, frequently leaving the patient or affected animal with a poor or fatal prognosis. Mechanisms that govern neuropathogenesis and immunopathogenesis of viral infections are highlighted, using examples of well-studied virus infections that are associated with these alterations in different populations throughout the world. A better understanding of the molecular, epidemiological and biological characteristics of these infections and in particular of mechanisms that underlie their clinical manifestations may be expected to provide tools for the development of more effective intervention strategies and treatment regimens.

**Electronic supplementary material:**

The online version of this article (doi:10.1007/s00401-015-1511-3) contains supplementary material, which is available to authorized users.

## Introduction

**Fig. 1:**
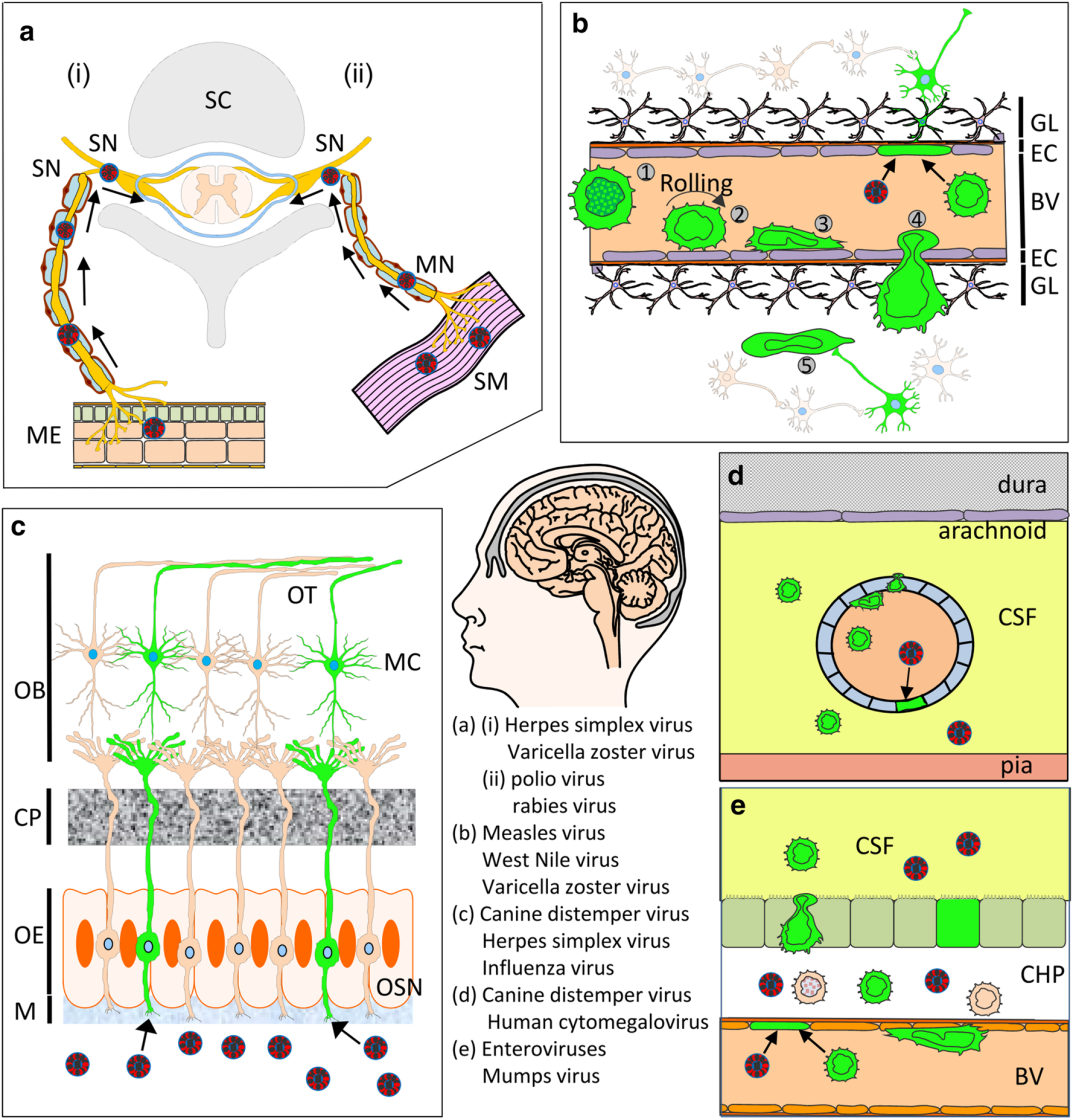
Routes of virus spread into the central nervous system. **a** Infection of peripheral nerves. (*i*) Virus spread from mucosal epithelium (*ME*) to sensory and autonomic neurons (*SN*) following infection of axon termini. Retrograde axonal transport results in virus spread to the spinal cord (*SC*). (*ii*) Virus infection of motor neurons (*MN*) at neuromuscular junctions in smooth muscle (*SM*) results in retrograde axonal transport to the spinal cord and the brain. **b** Blood–brain barrier (BBB). Virus-infected lymphocytes (*green*) (*1*) in blood vessels (*BV*) ‘roll’ along the endothelium (*2*), attach to the endothelial cells (*3*) and transverse the endothelial cell layer (*EC*) (*4*) and the glia limitans (*GL*). Virus spread to neurons (*5*) is assumed to occur following contacts with uninfected neurons. Alternatively, direct virus infection of endothelial cells may occur with subsequent spread into the brain parenchyma resulting in neuronal infection. **c** Infection of olfactory neurons. Virus present in the mucosa (*M*) of the upper respiratory tract can directly infect olfactory sensory neurons (*OSN*) present in olfactory epithelium (*OE*). Anterograde axonal transport leads to spread of virus within axonal bundles passing through the cribriform plate (*CP*) into the olfactory bulb (*OB*). Trans-synaptic spread to mitral cells (*MC*) results in virus spread along the olfactory tract (*OT*) to other brain regions. **d** Meningeal blood–cerebrospinal fluid (*CSF*) barrier. Virus-infected leukocytes in meningeal blood vessels present within the sub-arachnoid space between the pia and arachnoid roll, attach to the endothelium and transverse endothelial cells into the CSF. Direct infection of endothelial cells may also lead to virus spread into the CSF. **e** Blood–cerebrospinal fluid barrier. Virus-infected leukocytes or cell-free virus present within blood vessels of the choroid plexus (*CHP*) transverse the endothelium as described previously in **b**, **d**. This can lead to infection of epithelial cells and apical release of virus or spread of virus-infected leukocytes across the CHP epithelium into the CSF. Figure was composed using ©Motifolio.com Biomedical PowerPoint Toolkit Suite

Neurotropic virus infections continue to cause major disease and economic burdens on society, and pose a major challenge to human and animal health care systems due to the associated morbidity and mortality worldwide, and to the unique problems in providing treatment to the patients involved. This is largely due to unique features of the central nervous system (CNS), with a plethora of interconnected and interdependent cell types, complex structures and functions, reduced immune surveillance and limited regeneration capacity. Infection by neurotropic viruses as well as the local induced immune responses can irreversibly disrupt the complex structural and functional architecture of the CNS, frequently leaving the patient or affected animal with a poor or fatal prognosis. Besides immediate and direct effects, there are several neurological disorders often associated with autoimmune mechanisms that are assumed to be delayed virus-induced disorders: multiple sclerosis, Guillain–Barré syndrome, narcolepsy and encephalitis lethargica. Neurotropic pathogens can access the brain by various routes including retrograde axonal transport along motor and olfactory neurons, haematogenous spread across the blood–brain barrier (BBB), blood–cerebrospinal fluid barrier, meningeal–cerebrospinal fluid barrier, via direct infection of endothelial cells or via spread of infected leukocytes across the BBB into the brain parenchyma (Fig. 1). There is an unmet need to understand mechanisms that lead to neuropathological or immunopathological alterations occurring after the virus has entered the CNS or other parts of the body and the clinical manifestations that are associated with these changes. Furthermore, more insights into the molecular, epidemiological and biological characteristics of viral CNS infections are needed. Collectively, this will provide tools for the development of more effective intervention and antiviral treatment regimens. This development will be aided by the juxtaposition of increasingly sophisticated technologies, like those coming from the emerging fields of virus reverse genetics, brain imaging and advanced cellular phenotyping. This review aims to provide an updated overview of the different mechanisms involved in the pathogenesis of viral CNS infections, using clear examples of well-studied virus infections (Table 1), rather than by providing an exhaustive overview of the knowledge of all neurotropic viruses. For this reason we have not included any discussion of lentiviruses, many of which are also capable of infecting the CNS.

**Table 1:** Summary of virus-induced neurological disorders, diagnostic, therapy, pathology and virus-specific findings in humans

	Alphaviruses Bunyaviruses	Bornaviruses (VSBV-1)	Flaviviruses	Herpesviruses	Influenza virus	Picornaviruses	Paramyxoviruses	Rhabdoviruses
Virus characteristics	(+) ss RNA (alphavirus) (−) ts RNA (bunyavirus)	(−) ss RNA	(+) ss RNA	Large enveloped dsDNA	Segmented (−) ss RNA	(+) ss RNA	Non-segmented (−) ss RNA	Non-segmented (−) ss RNA
Clinical presentation	Encephalitis, meningitis, hemorrhagic manifestations	Encephalitis, myoclonus, ocular paresis	Encephalitis, meningitis,	Encephalitis, Meningitis, myelitis, polyradiculitis and neuropathies	Acute: varies from seizures to encephalitis/encephalopathy In utero: psychiatric disorders	Meningitis, encephalitis, poliomyelitis	Encephalitis, meningitis	Encephalitis, dysautonomia
Diagnosis	Clinical picture and IgM detection	Metagenomic analysis, RT-qPCR	IgM in CSF or serum neuroimaging	Clinical picture, neuroimaging and qPCR of CSF	Clinical picture, qPCR of CSF or post-mortem CNS tissues	RT-PCR CSF, stool, throat	Clinical picture, neuroimaging, IgG detection, RT-PCR CSF	Clinical picture RT-PCR, DFA detection
Pathology	VI	IP	VI and IP	VI: HSV and VZV IP: CMV, EBV and HHV6	VI and IP	VI	VI: MV, MuV, NiV, HeV IP: MV	VI
Specifics	Risk of sustained neurological sequelae	Fatal outcome; zoonotic infection	Patient age can affect prognosis Risk of neuropsychiatric sequelae	Risk factor for diseases such as vasculitis, neuralgia, limbic encephalitis, acute retinal necrosis		Acute infections with sometimes severe sequelae Chronic meningoencephalitis in hypogammaglobulinemia	Acute and persistent infections High fatality rate	Flaccid limb weakness invariably fatal outcome
Therapy	No antivirals supportive (IVIG)	No antivirals supportive (IVIG)	No antivirals supportive	ACV: HSV and VZV GCV and FC: CMV and HHV6	Antivirals, efficacy has not been proven	No antivirals Supportive (IVIG)	MV: Supportive (IVIG) MuV: Corticosteroids NiV: hMAb tested in AGM	Supportive (HRIG) Vaccine therapy No antivirals

*ACV* acyclovir, *AGM* African green monkey, *CHIK* Chikungunya, *CMV* cytomegalovirus, *CSF* cerebrospinal fluid, *dsDNA*, *DFA* direct fluorescent antigen; double-stranded DNA, *EBV* Epstein–Barr virus, *EEE* Eastern Equine encephalitis, *GCV* ganciclovir, *FC* foscarnet, *HSV* herpes simplex virus, *HeV* Hendra virus, *HHV6* human herpesvirus 6, *HRIG* human rabies immunoglobulin, *IP* immunopathology, *IVIG* intravenous immunoglobulin, *LACV* La Crosse virus, *MV* measles virus, *MuV* mumps virus, *NiV* Nipah virus, *qPCR* quantitative PCR, *RVFV* Rift Valley fever virus, *RT-PCR* reverse transcription polymerase chain reaction, *RT-qPCR* reverse transcription quantitative PCR, (+) *ss* positive-sensed single-strand, (−) *ss* negative-sensed single-strand, *TOSV* Toscana virus, *ts* three-segmented, *VEE* Venezuelan equine encephalitis, *VI* virus-induced, *VSBV-1* variegated squirrel 1 bornavirus, *VZV* varicella zoster virus

## Alphaviruses

The genus *Alphavirus* of the *Togaviridae* family comprises a group of enveloped, single-strand positive-sense RNA viruses, most of which are transmitted by mosquitoes. Typically, human infections with old-world alphaviruses such as O’nyong-nyong and Ross River virus manifest as fever, maculopapular rash and polyarthritis, whereas infection with the new-world equine encephalitis viruses (EEVs) can result in potentially fatal encephalitis. Encephalitic alphaviruses of public health concern include the new-world alphaviruses Eastern equine encephalitis virus (EEEV), Venezuelan equine encephalitis viruses (VEEV) and Western equine encephalitis virus (WEEV). More recently, the old-world Chikungunya virus (CHIKV) was also associated with encephalitis. Considering that WEEV cases have become infrequent and sporadic, this chapter focuses on neurologic manifestations of EEEV, VEEV and CHIKV infections.

EEEV is enzootic to the eastern United States, the Great Lakes region and the Gulf Coast where it circulates between birds and the ornithophilic mosquito *Culiseta melanura*. Several *Aedes*, *Coquillettidia* and *Culex* mosquitoes function as bridge vectors, transmitting the virus to equines and humans, which can suffer from severe disease. The related VEE viruses circulate in Central and South America. These viruses are grouped into six antigenic subtypes (I–VI). Human epidemics and equine epizootics are almost exclusively caused by viruses of antigenic variants A/B and C of subtype I. The remaining antigenic variants circulate in enzootic sylvatic cycles between rodents and *Culex* (*Melanoconion*) mosquitoes. Subtypes I A/B and C, which are highly virulent for horses, are believed to result from adaptation of enzootic strains to equines (horses, donkeys and mules). This adaptation allows the virus to replicate to extremely high levels, resulting in 20–80 % mortality [100]. *Aedes* (*Ochlerotatus*) *taeniorhynchus* is considered the major bridge vector of epidemic/epizootic strains. The geographic distribution of CHIKV includes sub-Saharan Africa, India, Southeast Asia, the Western Pacific with recent spread to the Caribbean and South America. The virus circulates in a sylvatic cycle between nonhuman primates and forest-dwelling *Aedes* mosquitoes. In the urban cycle, *Aedes aegypti* and *Aedes albopictus* mosquitoes are responsible for virus transmission to humans.

### Eastern equine encephalitis virus

EEEV infections in humans can manifest as two forms of disease: systemic or encephalitic. Systemic disease presents after an incubation period of 4–10 days as fever, malaise, muscle and joint pains and resolves without treatment within 1–2 weeks. The encephalitic form has an abrupt onset in infants, whereas in older patients neurological signs are observed a few days after onset of systemic disease. Patients may develop severe headache, confusion, neck stiffness, seizures, a decline in the level of consciousness and coma. Infection with the highly virulent North American (NA) EEEV strains can lead to a case fatality ratio of up to 40 %, which makes NA-EEEV the most deadly encephalitic alphavirus. Patients that survive the infection may suffer from serious sequelae such as mental retardation and paralysis.

Gross pathological examinations of human cases have revealed diffuse cerebral edema, vascular congestion and occasionally hemorrhages. Due to the hematogenous route of infection in the acute stage of the disease, a predominant panencephalitis and meningitis with vasculitis, occasional thrombosis and perivascular to widespread edema are found. Neuronophagia, microglial nodules and lympho-histiocytic, predominantly perivascular infiltrations are encountered in various regions of the brain. However, a predilection of damage exists for basal ganglia and brain stem. Alterations to the spinal cord are very limited with occasional lesions in the upper cervical region [28, 74, 117]. Neurons appeared to be the major target cells although infection of glial cells has been reported [41].

### Venezuelan equine encephalitis virus

Symptoms of VEEV infection generally manifest 2–5 days after a mosquito bite. Symptoms of VEEV infection include fever with chills, severe retro-orbital and occipital headache, nausea with vomiting, sore throat, diarrhea, tachycardia and myalgia; typically centering in the thighs and lumbar region of the back [139]. Children are more susceptible to severe disease than adults and are more likely to suffer from permanent neurological sequelae. Neurological complications include confusion, somnolence, delirium, nuchal rigidity, spastic paralysis, ataxia and photophobia. Less common symptoms include tremors, paralysis, nystagmus, pathological reflexes, cranial nerve palsies, syndrome of inappropriate antidiuretic hormone (SIADH) secretion, visual defects and coma. Lethal outcomes are associated with diffuse edema, brain hemorrhage, hepatocellular degeneration, alveolar hemorrhage and interstitial pneumonia [27, 62]. Although the overall case fatality ratio of VEE is below 1 %, its association with outbreaks involving tens of thousands of human cases renders it the most important encephalitic alphavirus.

Histopathology studies on autopsied patients have identified spleen, lymph nodes, intestinal lymphoid tissues, liver, lungs and the CNS as the principle target organs of VEEV. Moderate to marked diffuse congestion and edema with hemorrhage can be observed in brain, gastrointestinal tract and lungs. Mild or focal mixed inflammatory cell infiltrates can be detected in the leptomeninges and perivascular spaces. Depletion of lymphocytes with vascular thrombosis and necrosis of follicles can be observed in lymph nodes, spleen and gastrointestinal tract. Hepatocellular degeneration with necrosis of individual cells is observed in the majority of cases. A high percentage of individuals may present interstitial pneumonia. Although the major target cells of VEEV in humans have yet to be identified, pathological findings have suggested that hepatocytes, vascular endothelial cells, B cells, and cortical neurons are affected during the course of the disease. Importantly, lesions were proposed to result from direct injury or immune-mediated clearance of infected cells [27].

### Chikungunya virus

After an incubation period of typically 3–7 days, CHIKV infection manifests with high fever, headache, maculopapular rash and painful arthralgia. Other symptoms include retro-orbital pain, myocarditis and hemorrhage. Most patients recover within 1–2 weeks, but joint pain may persist for several months to years. Sporadic CHIKV neurological manifestations have been described since the 1960s and 1970s, but cases were not well documented. More recently, a series of serious outbreaks occurred in countries with modern clinical facilities that facilitated more detailed studies leading to novel insights into CHIKV-mediated neurological disease. During the well-documented outbreak in 2005–2006 on Réunion Island, neurological signs were reported in 12 % of patients [14]. In children and adults, neurological manifestations include altered levels of consciousness, cranial nerve deficits, seizures, decreased deep tendon reflexes, psychosis, hemi/paraparesis, paraplegia and involuntary movements. A subset of children of <1 year presented with hypotonia, tense fontanelle and status epilepticus [10]. Apart from mosquito-transmitted infection, CHIKV can be transmitted from viremic mother to child during birth. Although rare, these infections result in severe encephalopathy in almost half of the cases. Manifestations include brain swelling, disseminated intravascular coagulation, cerebral or cerebellar hemorrhage, scattered parenchymal petechiae, cerebellar hematoma and hematemesis [43]. Brain lesions comprise edema with focal ischemic changes in the frontal and occipital cortexes and the internal capsule, as well as hemorrhages, demyelination and cavitations located in the periventricular subcortical white matter. Minimal microgliosis may occur in the cortical gray matter and diencephalon with occasional perivascular lymphocytic infiltrates noted [40]. The changes seem to result from transient ischemia with cytotoxic edema and not from neuronal death.

## *Bornaviridae*

Bornaviruses belong to the family *Bornaviridae,* of the order *Mononegavirales* and are single-stranded negative-sensed RNA viruses. These viruses display a number of unique features that are not typical for non-segmented negative-sense RNA viruses such as nuclear transcription and replication and their ability to establish a non-cytolytic persistent CNS infection. The classical mammalian Bornavirus BoDV-1 (Borna disease virus, BDV), the prototype of this family, causes a typically fatal neurological disease (Borna disease, BD) in horses and sheep in endemic areas of central Europe with an incubation period of 2–4 months before the onset of clinical neurological signs of infection (reviewed in [55]). However, in experimental settings, the host range is much wider, ranging from chickens to primates. Serological studies have shown that the distribution of Bornaviruses might be widespread, possibly worldwide. However, clinical BD accumulates in geographically restricted endemic areas, suggesting the existence of a natural reservoir. In accordance to this, infectious BoDV-1 was detected in tissues of white-toothed shrews (*Crocidura leucodon*) in endemic areas of Germany and Switzerland [15, 56].

BoDV-1 was considered the only member of the family *Bornaviridae* until the last decade. Since then, knowledge on Bornaviruses has expanded tremendously. Endogenous genome-integrated Bornavirus-like sequences have been detected in various vertebrate species, including snakes, bats, elephants, fish, lemurs, rodents, squirrels, primates and humans [38, 59]. Moreover, new genetically distant Bornaviruses have been detected in many other species, including psittacine birds, water fowl, and reptiles by means of next-generation sequencing [58, 67]. A novel Bornavirus, recently discovered in variegated squirrels (variegated squirrel Borna virus-1; VSBV-1), was associated with three human cases of fatal encephalitis indicating its zoonotic potential [57]. In contrast, the serological and virological data for human infections by BoDV-1 are more uncertain. BoDV-1-specific antibodies can be present in sera of patients with various psychiatric conditions but also in clinically healthy people. There is general consensus that BoDV-1 is not associated with human psychiatric illness [34, 60]. It should be emphasized that the human VSBV-1 infections (encephalitis) are quite different from previous studies dealing with potential human BoDV-1 infections [57]. The incubation period of VSBV-1 or potentially BoDV-1 in humans is unknown.

**Fig. 2:**
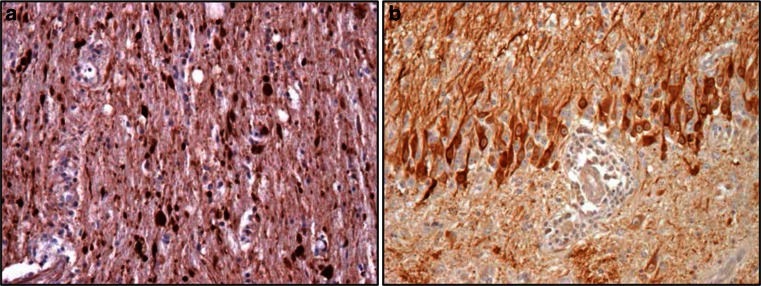
Immunohistochemcial detection of the X-protein of novel Bornavirus (variegated squirrel Borna virus-1; VSBV-1) in human brain sections (**a**) and demonstration of Borna disease virus 1 (BoDV-1) X-protein in the hippocampus of a horse which had neurological signs of BoDV-1 infection (**b**). Viral X-protein is present in the nuclei, cytoplasm and cellular processes of infected neurons

In the natural host, *Crocidura leucodon*, BoDV-1 disseminates to both neural and extra-neural tissues and is continuously shed via secretions, excretions and skin scaling [15, 92]. In contrast, BoDV-1 infection is strictly neurotropic in dead-end hosts, in which it causes a severe non-purulent meningoencephalitis. The newly detected VSBV-1 was also found in many organs including the CNS in a contact squirrel and brain of diseased squirrel breeders (Fig. 2a) who also presented with non-purulent (meningo-) encephalitis [57]. Patients suffered from shivers, fever, confusion, psychomotor and gait disturbances and finally coma. All of them had underlying medical conditions such as hypertension, diabetes or obesity. Infection of horses with BoDV-1 may manifest as excited or depressed behavior, abnormal posture and movement and may result in a mortality rate of up to 90 %. However, in the majority of equine cases, BoDV-1 infections are clinically inapparent and are associated with long-lasting specific serum antibodies.

Experimental Bornavirus infections have provided evidence for intranasal virus transmission via olfactory nerve endings with retrograde transaxonal spread to the olfactory bulb and subsequently the brain. Primary target cells are neurons and astrocytes, but oligodendrocytes and ependymal cells can also be infected. Pathogenesis is best studied in the rat model where remarkable differences in clinical course and virus distribution occur depending on the state of the immune system and time point of infection (reviewed in [55]). Infection of newborn immunocompetent rats results in a persistent CNS infection with virus spread to peripheral organs with no obvious neurological signs. However, disturbances in learning and memory and loss of distinct hippocampal and cerebellar neurons are observed. In adult rats, virus infection is also persistent but it is restricted to the CNS and associated with a severe non-purulent meningoencephalitis and biphasic neurologic disease comparable to clinical signs observed in humans and equines. In horses and rats, the characteristic histological picture consists of extensive mononuclear perivascular cuffing, astroglial and microglial activation and eosinophilic intranuclear inclusions in neurons, called “Joest–Degen” bodies with viral antigen detected in immunohistochemically stained brain sections (Fig. 2b). In VSBV-1-infected patients, comparable lesions develop with edema, necrosis, glial activation, and lymphocytic, often perivascular infiltrates. In animals, gray matter areas are predominantly affected, e.g., cerebral cortex, caudate nucleus, thalamus and hippocampus. Experimental BoDV-1 infection of rodents implicates a pivotal role of immunopathogenic CD4 and CD8 T cells (reviewed in [55]). Thus, infection does not cause a protective immune response and virus-specific antibodies typically lack virus-neutralizing capacity.

## *Bunyaviridae*

The family *Bunyaviridae* comprises over 350 named viruses, most of which are transmitted by insects or ticks. Bunyaviruses are enveloped, spherical particles of about 100 nm in diameter, containing a three-segmented negative-strand RNA genome. The Orthobunyavirus and Phlebovirus genera include members that are associated with human CNS disease. The Orthobunyavirus genus is the largest of the *Bunyaviridae* family, comprising more than 170 viruses. Important human pathogens include Oropouche virus, Ngari virus and La Crosse virus (LACV); whereas the former two are associated with acute, self-limiting febrile illness or hemorrhagic fever, respectively, LACV is associated with neurological disease and is the major cause of arboviral (arthropod-borne virus) neuroinvasive disease in the United States. LACV is present throughout the mid-western and eastern United States and is also emerging in Appalachia, where it circulates between the primary mosquito vector *Aedes* (*Ochleratatus*) *triseriatus* (eastern treehole mosquito) and small mammals, mostly chipmunks and tree squirrels. A member of the phlebovirus genus associated with encephalitis is Rift Valley fever virus (RVFV). RVFV is transmitted by *Aedine*, *Culicine* and *Anopheline* mosquitoes among mammals, of which domesticated ruminants are the most susceptible to disease. Currently, the virus is largely confined to the African continent, but global distribution of potential mosquito vectors explains the fear for future “virgin soil” epizootics and epidemics. The sandfly-transmitted Phlebovirus, Toscana virus (TOSV), is associated with neurological disease and is a major cause of acute, generally mild aseptic meningitis in the Mediterranean region. In these areas, the virus is transmitted by *Phlebotomus perniciosus* and *Phlebotomus perfiliewi* flies.

### La Crosse virus

Symptoms resulting from LACV infection develop after an incubation period of 5–15 days. Like most arbovirus infections, LACV infections are generally subclinical or present as a self-limiting febrile disease with headache, fever, nausea and vomiting with minimal neurological involvement. Children under 15 years of age are at risk of developing severe neurological disease, which may resemble herpes simplex virus (HSV) encephalitis, particularly when focal signs are present [79, 148]. Red blood cells and increases in protein concentrations may be detected in cerebrospinal fluid (CSF), although not as pronounced as in HSV encephalitis. Symptoms include focal neurological signs, photophobia, decreased alertness, drowsiness, neck stiffness and disorientation. Up to 50 % of hospitalized patients develop seizures. Case fatality rate among symptomatic cases is below 1 %, whereas a high case fatality rate of 3.1 % was recorded in West Virginia [50]. Pathology in humans is believed to result from infection, dysfunction and death of neurons, although in vitro studies demonstrated that LACV infection does not result in apoptosis of postmitotic human neurons [97]. The role of neurons in LACV pathogenesis as well as those of additional target cells thus remains to be elucidated. Children are predisposed to LACV-induced CNS manifestation and may develop cerebral edema with increase of intracranial pressure. Morphological changes comprise lymphocytic leptomeningitis and perivascular cuffs, occasional foci of necrosis, predominantly in the cerebral cortex and brain stem [74].

### Rift Valley fever virus

Human RVFV infections result from mosquito bites or contact with contaminated animal products. Most infections manifest typically after 2–6 days as a self-limited, benign illness with flu-like symptoms. In <1 % of cases, severe complications may develop including fulminant hepatitis, renal failure, retinopathy resulting in temporary or permanent blindness, hemorrhagic fever and encephalitis. The latter presents 1–4 weeks after onset of initial symptoms. The first fatal RVF cases, associated with hemorrhagic fever and encephalitis, occurred in South Africa in 1975 [134]. Encephalitis cases occurred during all major subsequent RVF outbreaks, although the incidence among hospitalized patients varied greatly from <10 to 89 % [84]. CNS manifestations included meningeal irritation, confusion, hypersalivation with teeth-grinding, hallucinations, lock-in syndrome, choreiform movements, stupor and coma. Histopathological examinations of the brain of fatal cases have revealed focal areas of necrosis associated with lymphocyte and macrophage infiltration [134] or mild congestion and edema of the white substance with mild hypoxic degeneration of cerebral neurons [1]. However, further studies are required to identify the target cells of RVFV in the human CNS. Studies with nonhuman primates have provided important insights into RVFV-mediated neuropathogenesis. The most robust RVF nonhuman primate model makes use of common marmosets (*Callithrix jacchus*). In these monkeys, disease following peripheral infection manifests as either severe hepatic/hemorrhagic disease or encephalitis, whereas intranasal inoculation results in fatal encephalitis [119]. In a subsequent study, aerosol exposure of common marmosets but also of African green monkeys resulted in fatal encephalitis [53]. Primary pathology in the brain was associated with apoptosis of neurons in many regions of the brain with limited or no inflammatory response.

### Toscana virus

The high seroprevalence of TOSV antibodies in the Mediterranean region with limited reports of clinical cases makes clear that TOSV infections are generally asymptomatic or manifest as a self-limited benign illness. This so-called “sandfly fever” has an incubation period of 3–6 days and may be associated with fever, myalgia, malaise and abnormalities in liver and hematological values. A substantial number of patients develop skin rash. Neurological manifestations may present with Kernig sign, nuchal rigidity, photophobia, consciousness troubles, tremors, nystagmus and paresis. In some patients, the disease progresses from mild acute lymphocytic meningitis to meningoencephalitis or even encephalitis [29]. Although most patients recover within 7–10 days without sequelae, sporadic life-threatening atypical manifestations of TOSV infection have been reported [11]. Because TOSV infections are generally benign, very little is known about the pathogenesis of this virus in humans.

## *Flaviviridae*

The family *Flaviviridae* consists of four genera; *Hepacivirus* which includes Hepatitis C virus, *Pestivirus* which includes Bovine viral diarrhea virus, *Pegivirus* which includes GB virus C (formally hepatitis G virus) and the genus *Flavivirus*. The *Flavivirus* genus includes viruses that are etiological agents of arboviral encephalitides and more systemic disease spectra including hemorrhagic fevers (e.g., dengue virus and yellow fever virus). Flaviviruses are single-strand positive-sense RNA viruses.

### Tick-borne encephalitis virus

**Fig. 3:**
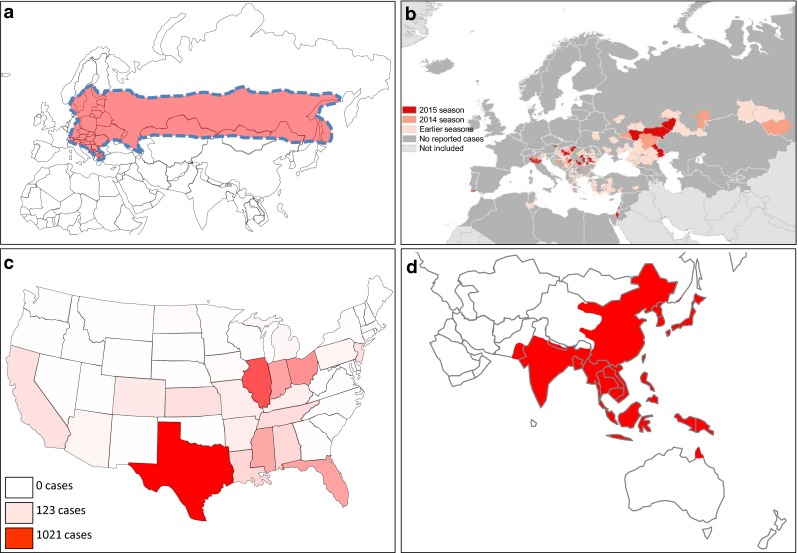
Geographical distribution of selected flaviviruses capable of causing neurotropic infections in humans. **a** Cases of tick-borne encephalitis (demarcated within *red*-*shaded zone*) are distributed from central and eastern Europe across a wide band of Eurasia. **b** Distribution of cases of West Nile fever in the European region and Mediterranean basin in 2015 and previous seasons. **c** Gradient map showing cumulative cases of St. Louis encephalitis virus neuroinvasive disease in the USA from 1964 to 2010. **d** Cases of Japanese encephalitis virus (JEV) (*red*-*shaded area*) are diagnosed in many parts of South East Asia. **a** Adapted from [73]; **b** adapted from European Center for Disease Control and Prevention (ECDC) map of reported cases of West Nile fever, transmission season 2015 and previous transmission seasons; **c** adapted from Centers for Disease Control and Prevention (CDC) map; **d** adapted from CDC map of geographical distribution of Japanese encephalitis virus

Tick-borne encephalitis virus (TBEV) is an important cause of CNS infection in Europe and Asia [77]. The disease is reported over a wide geographical range, spanning from Japan, throughout Asia into Europe (Fig. 3a). Thousands of human cases of TBEV infection are reported annually in Europe and Asia. The virus is transmitted by the ticks *Ixodes ricinus* and *Ixodes persulcatus* in Europe, whereas *Ixodes ovatus* transmits the virus in Japan. Other virus transmission modes include consumption of infected unpasteurized milk or milk products from infected livestock, particularly goats. Phylogenetic analysis of several TBEV strains indicated the existence of three subtypes: (1) European (2) Siberian and (3) Far-Eastern subtype. The differences between the subtypes constitute 5–6 % on amino acid level, but it is unclear whether these subtypes differ in virulence.

The incubation period of TBE depends on the route of infection, which ranges from 7 to 14 days after a tick bite and 3–5 days after alimentary transmission [109]. About 70 % of TBEV infections are asymptomatic and only a few will develop mild flu-like disease. In general, the course of TBEV disease is mono- (up to 30 %) or biphasic (>70 %). The prodromal phase correlates with high viremia and is characterized by flu-like symptoms such as fever, headache, myalgia, arthralgia, fatigue, anorexia and nausea. After 2–7 days, symptoms subside and patients may even recover. This brief recovery period is followed by the second disease phase where adult patients may develop meningitis (50 %), meningoencephalitis (40 %) or meningoencephalomyelitis (10 %) [65]. Most patients with the monophasic disease course develop more severe acute disease, presenting with meningitis or meningoencephalitis. A small fraction of patients with CNS involvement have significant variation in heart rate or display signs of autonomic nervous system dysfunction. Chronic progressive TBE has been reported in Siberia and the Russian Far East, which is believed to be caused by the Siberian TBEV subtype. TBE may cause long-lasting impact in patients’ quality of life. The most frequently reported problems are cognitive disorders, neuropsychiatric complaints hearing loss, disturbances of vision, balance and coordination disorders and flaccid paresis or paralysis.

**Fig. 4:**
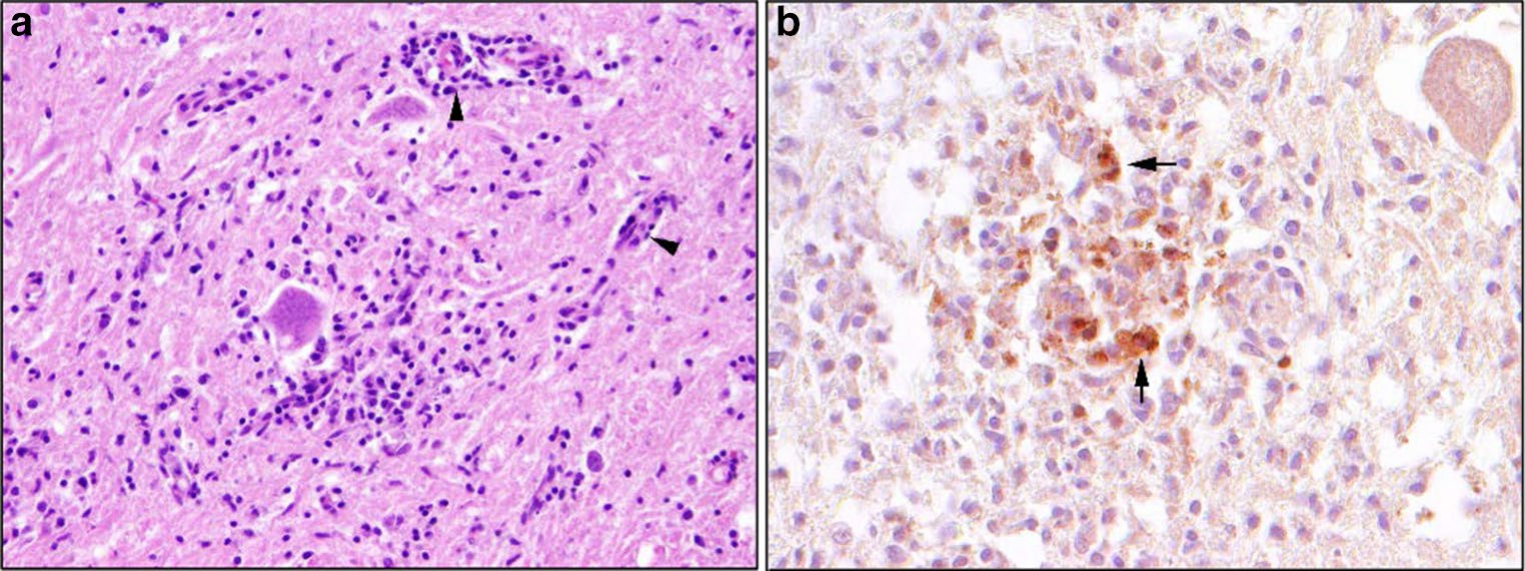
Neuropathology in the spinal cord of a 3-year-old dog (Bernese mountain dog) infected with tick-borne encephalitis virus (TBE). **a** Hematoxylin and eosin-stained section showing severe lympho-histiocytic myelitis with perivascular predominantly lymphocytic cuffing (*arrowheads*). **b** Intralesional immunolabelling of TBE antigen (*arrows*), detected using a rabbit polyclonal antibody

The frequency of abnormal electroencephalogram findings appeared similar between patients with meningoencephalitis (91 %) and meningoencephalomyelitis (96 %) [65]. MRI data suggest that patients with meningoencephalitis have lesions predominantly in the thalamus. Lesions have also been reported in the cerebellum, brainstem and caudate nucleus. Immunohistochemical analysis demonstrated TBEV protein expression in the spinal cord, brainstem, cerebellum and basal ganglia [42]. A similar widespread distribution of lesions and virus antigen can be detected in TBEV-infected dogs (Fig. 4). Analysis of human brain tissues has shown consistent detection of virus antigen in neurons in the anterior horn, Purkinje cells, dentate nucleus, *tegmentum* of *medulla oblongata* (e.g., *inferior olives*, raphe nuclei and *formatio reticularis*), pontine nuclei and tegmentum of pons (*L. coeruleus*, *N. dorsalisn. vagi* and *formatio reticularis*) and caudate nucleus [42].

### West Nile virus

West Nile virus (WNV) is transmitted between birds by *Culex* mosquitoes, in an enzootic cycle. Humans become infected when bitten by a mosquito carrying the virus. Since the first isolation of WNV in 1937 there have been sporadic cases and outbreaks worldwide. A number of large outbreaks have occurred in North America since 1999 including a particularly large outbreak of encephalitis in 2002 with 2942 cases [93] with additional smaller outbreaks occurring annually in Southern and Eastern Europe (Fig. 3b). On average 1 in 140 infected individuals will develop meningoencephalitis, but the incidence is higher in the older population (1 in 50 in >65 years compared to 1 in 300 in <65 years old). There are seven genetic lineages of the virus. Lineages 1 and 2 are responsible for the major epidemics in humans and animals. It is thought that migratory birds contributed to the worldwide spread of WNV such as from Africa to Southern Europe. However, this hypothesis has been challenged with respect to the introduction of WNV to North America as it is unlikely that sick birds are capable of flying the required distances for this route of transmission to be viable. An alternative hypothesis is that mosquitoes were the cause of virus importation into North America.

The incubation period for WNV is normally 3–14 days. Patients with WN fever develop a sudden onset of an acute non-specific flu-like illness [123]. Some patients may also develop maculopapular rash, but myocarditis has also been described. Among individuals with CNS infection, presentation with either encephalitis or meningitis may be seen. However, the elderly more commonly present with encephalitis [25]. Severe generalized muscle weakness with a similar presentation to Guillain–Barré syndrome has been reported probably as the result of damage to the anterior horn cells [123]. An acute flaccid paralysis (AFP) was also seen in patients during a few outbreaks [5]. Seizures were seen in approximately 30 % of patients during earlier outbreaks but not during more recent ones. Mortality ranged from 2 to 14 % during the outbreak in North America with elderly people having the greatest risk of death [123]. Neurological disability was seen in just over half of patients at 1-year follow-up. There is no consensus on how WNV enters the CNS. Theories include direct infection of endothelial cells, infecting leukocytes which carry the virus into the CNS, crossing endothelial tight junctions, direct axonal retrograde transport from infected peripheral neurons. WNV infection is characterized by lymphocytic leptomeningitis, perivascular lymphocytic cuffs and lymphocytic polioencephalomyelitis. Numerous microglial nodules and astrogliosis may occur in both the gray and white matter and involve the entire neuraxis. In the gray matter, microglial nodules are often associated with neuronophagia and neuronal loss. Several brain regions, including the cerebral cortex, hippocampus, cerebellum, brainstem and spinal cord are particularly affected [66, 115]. It is still unclear as to how the virus causes neuronal injury, but most likely several mechanisms may contribute. While it has been suggested that apoptotic cell death of WNV-infected neurons is caspase-3 dependent [110], in vitro studies indicate that caspase-3-independent pathways also exist in WNV-associated cell death. In addition, infection of neurons with WNV results in the induction of several cytokines and chemokines, which promote leukocyte invasion into the CNS and neuroinflammation [68]. Furthermore, there is evidence that WNV also infects glial cells, and that infection of astrocytes contributes to neuronal death by releasing neurotoxic mediators [131]. MRI imaging may show atrophy or ischemic changes as well as meningeal enhancement [91]. T2-weighted images have also shown high signal intensities in the thalamus and other basal ganglia [121].

### St. Louis encephalitis virus

St. Louis Encephalitis virus (SLEV) is found in the Americas with eight recognized lineages [106]. The virus is transmitted by *Culex* mosquitoes to vertebrate hosts including birds and bats [7, 69]. SLEV-mediated encephalitis occurs following an incubation period of 5–15 days and is seen in approximately 50 people per year in North America with 800 cases per 100,000 population occurring during epidemics which have a localized geographical distribution (Fig. 3c). Patients present with flu-like symptoms and in some cases diarrhea, nausea, vomiting, cough and sore throat may also occur. In individuals >60 years old, 90 % will develop encephalitis whereas this is only seen in 55 % of the younger patient. In the latter group the most common clinical manifestation is a reduction in conscious level. Cranial nerve palsies and urinary tract symptoms may also occur [124]. Seizures are a poor prognostic sign and can be seen children and more severe adult cases. One-third of patients have neuropsychiatric sequelae including emotional disturbances, forgetfulness, tremor, unsteadiness and visual disturbances. Death occurs in approximately 7 % of patients with higher fatality rates in the elderly. Neuroimaging of patients infected with SLEV has consistently shown the presence of lesions in the substantia nigra [21, 137], a finding which may explain the tremors that are often observed in symptomatic cases. Neuropathology investigations have shown that pathological changes are largely restricted to the substantia nigra and cerebellum with neuronal degeneration, microglial activation and perivascular infiltrates of mononuclear cells observed in brain sections from these regions [104, 137]. Although SLEV antigen has been detected in the cell bodies of neurons [104], few studies have specifically examined the distribution of this virus in the CNS of fatal human cases.

### Japanese encephalitis virus

Among the many arthropod-borne viruses that cause brain infections, Japanese encephalitis virus (JEV) is the most important, being responsible for large outbreaks across Asia. JEV is endemic in South and Southeast Asia (Fig. 3d). It is transmitted by *Culex* mosquitoes, most commonly *Culex tritaeniorhynchus*, which breed in rice paddy fields and other water sources. Virus transmission occurs in an enzootic (animal) cycle with a natural transmission cycle between birds and mosquitoes and an ‘amplifying cycle’ between pigs and mosquitoes. JEV can be transmitted to humans via the bite of an infected mosquito which subsequently becomes the ‘dead-end’ host with no further transmission of the virus. It is estimated that <1 % infected individuals develop clinical features following an incubation period of 5–15 days, which may range from a flu-like illness, to severe and often fatal encephalitis in ~20 % of patients. Whereas children are more commonly affected, JEV-induced disease is also seen in adults, especially in areas where there is no pre-existing immunity. Seizures are a frequent clinical manifestation. However, AFP, extra-pyramidal features and focal neurological deficits have also been described. Those with a reduced conscious level or abnormal tone, signs of brainstem herniation, raised intracranial pressure, seizures and fever tend to have a worse prognosis [120]. Disability is seen in up to 50 % of survivors, often with a hemiparesis [102]. However, seizures, behavioral problems and language impairment have also been described [49, 94].

Upon skin inoculation, the virus spreads hematogenously to the liver, kidneys, heart and spleen. The virus eventually crosses the blood–brain barrier (BBB) to enter the CNS. There is still uncertainty as to how the virus crosses the BBB. One theory is a ‘Trojan horse’ mechanism, whereby the virus is transported across the BBB via macrophages or monocytes. Other suggested theories include the passive transport of the virus across the endothelium, [35, 123] and immune-mediated damage to the BBB due to various proinflammatory cytokines. Compared to JEV survivors, cytokine levels in serum and CSF are increased in fatal cases [141]. High signal intensity in the thalamus, and other basal ganglia, midbrain, brainstem, and sometimes anterior spinal cord may be seen on T2-weighted MRI scans. Grossly the brain is swollen and congested. Lymphocytic infiltration is present throughout the brain, but particularly affected are the gray matter of the thalamus, substantia nigra, pons, medulla and spinal cord. Occasionally necrotizing encephalitis is encountered. Viral antigen is located in neurons of the cerebral cortex, thalamus, and brain stem. Glial scars may occur in substantia nigra and thalamus, and to a lesser extent in the cerebral cortex in long-term survivors [74]. There have been various suggestions as to why some people develop clinical manifestations whereas others do not. This may be attributed to the viral strain itself [23] or prior infection with other flaviviruses, including dengue virus, which may give a cross-protective immune response that may lead to more severe immunopathology [72, 123].

### *Herpesviridae*

The *Herpesviridae* is an ancient family of enveloped double-stranded DNA viruses which are widely disseminated in the animal world. Based on genomic and biological properties herpesviruses are divided into three subfamilies: α, β and γ viruses. Alpha-herpesviruses have a short reproductive cycle and establish latency in sensory ganglion neurons. Beta- and gamma-herpesviruses have a more restricted cell type range and replicate relatively slow in cell culture. Whereas beta-herpesviruses can establish latency in multiple cell types and organs (e.g., secretory glands, lymphocytes and kidneys), gamma-herpesviruses are restricted to specific lymphocyte subsets including B and T cells. Among the eight human herpesviruses (HHV), the alpha subfamily includes herpes simplex viruses type 1 (HSV-1) and -2 (HSV-2) and varicella zoster virus (VZV). Human cytomegalovirus (HCMV) and the roseola viruses HHV-6 and -7 belong to the beta subfamily and the oncogenic gamma subfamily consists of Epstein–Barr virus (EBV) and HHV-8. Primary HHV infection, commonly during early childhood via the orofacial route, is typically asymptomatic or mild (e.g., varicella and roseola infantum due to VZV and HHV-6, respectively) and does not warrant therapy [98].

A hallmark of herpesviruses is the establishment of life-long latency in specific cell types and intermittent reactivation leading to asymptomatic virus shedding or recrudescent disease. Whereas innate immunity prevents virus dissemination during primary infection, adaptive immunity is pivotal to control latency. Consequently, severe HHV infections are more common in HHV-naïve adults and immunocompromised patients, but also associated with the anatomic site of infection. HHV infections of immune-privileged sites, like the CNS and eye, may lead to potential sight- or even life-threatening diseases, respectively. Whereas HSV and VZV enter the CNS via the transaxonal route, the remaining six HHVs spread to the brain via the hematogenous route within lymphocytes. The high prevalence of HHV infections worldwide argues for their potential etiopathogenic role in CNS diseases that can be monophasic, recrudescent or even chronic. The clinical and pathological features of the key HHVs associated with CNS dysfunction will be discussed.

### Herpes simplex virus

**Fig. 5:**
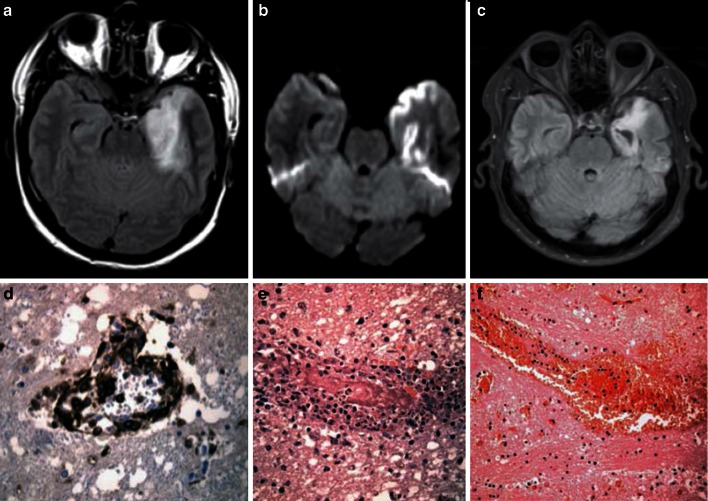
Magnetic resonance imaging and microscopic images of typical herpes simplex virus (HSV) encephalitis pathology in humans. Magnetic resonance imaging (MRI) of an acute HSV encephalitis case. **a** Swelling of the left temporal lobe (Fluid attenuation inversion recovery, FLAIR). **b** Cortical cytotoxic edema typical for this neurotropic virus at the time of symptom onset (diffusion-weighted image, reduced apparent diffusion coefficient values not shown). **c** Parenchymal atrophy with residual gliosis 2 months later (FLAIR). Microscopic imaging of autopsy material of the basal ganglia (**d**, **f**) and the frontal lobe (**e**) of a patient with fulminant HSV encephalitis. **d** Immunohistochemically stained sections showing CD45-positive perivascular infiltrates. **e** Hematoxylin and eosin staining (H&E) showing additional intravascular thrombosis. **f** Several parenchymal hemorrhages in an H&E-stained brain section. **a**–**c** Images courtesy of Dr. Peter Raab (Department of Neuroradiology, Hannover Medical School, Hannover, Germany); **d**–**f** Images courtesy of Prof. Christian Hartmann (Department of Neuropathology, Hannover Medical School, Hannover, Germany)

HSV infection, either due to primary or reactivation of latent virus, is the most common cause of infectious encephalitis in humans [24]. The incubation period of HSV-1 following primary infection is 2–12 days whereas HSV-2 has an incubation period of 3–7 days in susceptible individuals. HSV-1 is responsible for up to 90 % of HSV encephalitis (HSE) cases, whereas HSV-2 infection being less common and more often presenting clinically as meningitis. Predisposing factors are diabetes mellitus, malignancies and conditions compromising the immune system. Interestingly, defects in the TLR3-interferon (IFN) and IFN-responsive pathways were shown to predispose to HSV encephalitis, particularly in children [150]. Therefore, TLR3-mediated immune response in the CNS may play an important role to control local HSV infection. Clinically, HSE patients present with headache, fever and alteration of mental status such as confusion, psychosis and also alteration of consciousness from somnolence to stupor and coma. With a predilection of HSV for the frontal and temporal lobes, HSE patients may also present with seizures. In immunocompromised patients, HSE can be atypical and the disease may evolve rapidly and is life-threatening [81]. MRI scans of HSE patients often show hyperintensities on flair and T2-weighted images corresponding to cytotoxic edema in these areas (Fig. 5a–c).

Clinical outcomes are variable and largely depend on prompt initiation of antiviral therapy. Prior to use of the antiviral acyclovir (ACV), mortality of HSE patients was >70 % with a high rate of severe sequelae among survivors. Sequelae are still found and include focal or generalized seizures, personality changes, impaired memory and/or cognition, motor deficits or aphasia. Predictors for a prolonged clinical course are the initial clinical presentation with coma and high lesion load on brain imaging, but not duration of antiviral therapy or age at onset of disease [128]. HSE is the most common fatal sporadic encephalitis. Necrotizing encephalitis is commonly localized to the orbitofrontal and temporal lobes, usually asymmetrical, but in most cases bilateral. Progressive temporal lobe edema can lead to uncal herniation [126]. HSV-1 infections can also cause a myelitis presenting as subacute or chronic monosegmental myelitis. Re-occurrence of HSV myelitis has been reported in up to 20 % of cases. Neonatal HSV infection is mainly caused by HSV-2, due to mother-to-child transmission, with CNS involvement in about half of the infected infants [126]. Morphologically, acute ascending necrotizing myelitis occurs throughout the whole length of the spinal cord and involves both the gray and white matter [61]. Risk of long-term complications is high despite prompt ACV treatment. Sacral and lumbar radiculitis (Elsberg syndrome) has also been associated with HSV-2 infections [36]. Recurrence of meningitis is common in HSV-2 meningitis, and if large endothelial cells termed “Mollaret cells” are present in CSF analysis, the diagnosis of Mollaret’s meningitis is established. Pathology of HSV-induced CNS disease is largely due to viral cytopathic effect (Fig. 5d–f). HSV primarily infects neurons showing intranuclear inclusions upon biopsy or autopsy. Phagocytosis of neurons by microglia, so-called neuronophagia, may occur. Recently, the occurrence of an autoimmune antibody-mediated limbic encephalitis (NMDAR-encephalitis) has been associated with a history of HSV encephalitis [9]. HSE has also been described as a risk for subsequent potential blinding acute retinal necrosis (ARN) [135]. These complications can occur independently of the immune status. Therefore, a close and careful clinical follow-up of HSE patients is recommended to early identify potentially life-threatening and disabling subsequent diseases.

### Varicella zoster virus

VZV, a neurotropic HHV that establishes life-long latency in almost all sensory ganglia, is the only HHV that causes two different diseases during primary (chickenpox: varicella) and reactivation (shingles: herpes zoster) [98]. Varicella is evident 14–16 days following primary VZV infection. VZV-induced neurological disease is rarely associated with chicken pox (varicella cerebellitis), but commonly after shingles involving the CNS (meningitis and myelitis) and peripheral nervous system (cranial neuropathies and motor radiculopathies). Immunocompromised individuals are at increased risk of VZV-induced neurological disorders, particularly encephalitis. Cranial nerves, particular trigeminal and facial nerves, can also be affected and present as zoster ophthalmicus and facial nerve palsy, respectively [45]. The main complication is post-herpetic neuralgia (PHN), a neuropathic pain syndrome that may persist for months within the affected skin when shingles has already subsided. PHN pathology is most likely due to damage of the sensory nerve endings due to persistent VZV infection in the innervating ganglia and/or local inflammatory responses. Prognosis of VZV-induced CNS disease is better than for HSV-induced CNS disease. But unlike HSV, VZV can infect vascular endothelial cells of large and small cerebral vessels leading to severe focal or multifocal brain ischemia, vessel wall necrosis with aneurysms and dissections [90]. A particular association of VZV vasculitis and zoster ophthalmicus has been reported [16]. Likewise HSV, VZV encephalitis can be followed by ARN (see above). In situ analyses on biopsy and autopsy specimens showed that VZV infection can be found in neurons, glial cells and infiltrating macrophages. In contrast to HSV, VZV infections in immunocompromised patients may induce multifocal encephalitis, ventriculitis, focal necrotizing myelitis, acute myeloradiculitis, cerebral infarcts and macrophage-rich demyelinating “multiple sclerosis-like” periventricular cerebral and spinal cord lesions [89], probably due to an infection of oligodendrocytes. Intranuclear inclusions, viral antigen, and herpesvirus particles have been detected in ganglia of acutely infected patients [89]. The presence of infiltrating lymphocytes in affected nervous tissue indicates the additional involvement of the immune system in the pathology of VZV-induced neurological disorders.

### Cytomegalovirus

Human cytomegalovirus (HCMV) has the largest genome amongst HHV and causes a variety of diseases, ranging from self-limiting to fatal [98] with clinical symptoms evident in some patients 3–12 weeks following infection. HCMV-induced neurological disorders, mainly in immunosuppressed patients due to HCMV reactivation, include lumbosacral polyradiculomyelitis, longitudinal extensive transverse myelitis and encephalitis [113]. Before the advent of ART, HCMV was the most frequent opportunistic CNS infection in AIDS patients. HCMV neurological diseases, particularly encephalitis, are difficult to diagnose because of atypical clinical and neuroimaging features. Studies on HCMV-affected brain tissues implicate that the basal ganglia, diencephalon and brainstem are the major sites of HCMV infection. In situ analyses showed that the majority of cytomegalic cells originate from infected astrocytes, but HCMV has also been detected in neurons, astrocytes, oligodendrocytes, ependymal, choroid plexus and endothelial cells. HCMV neuropathology may present as low-grade encephalitis with widespread microglial nodules often associated with few cytomegalic cells or as necrotizing ventriculoencephalitis with abundant intranuclear inclusion bodies [74]. Congenital HCMV infection is a major public health concern that causes severe neurological disease in infants leading to mental retardation, cerebral palsy and sensorineural hearing loss.

### Epstein–Barr virus

Epstein–Barr virus is the causative agent of infectious mononucleosis (IM) in naïve children and young adults, with clinical symptoms evident 4–6 weeks following infection. Less than 5 % of primary EBV infections cause CNS disease that present as meningitis, encephalitis, cerebellitis, cranial or peripheral neuropathies and polyradiculomyelitis. Neurological involvement may occur shortly before, during or after IM or even in the absence thereof. Polyradiculomyelitis is most often as a post-infectious autoimmune-mediated syndrome and patients respond well to steroid therapy often given in combination with antivirals [22]. EBV-related CNS diseases can be induced by primary infection or reactivation, but also associated with chronic EBV infection. In contrast to HSV and VZV, human EBV encephalitis lesions do not show presence of viral protein and nucleic acids questioning the direct neuropathic effect of the virus. Cerebellum and basal ganglia are reported to be equally involved during EBV infection, next to cerebral hemisphere. Patients with isolated hemispheric gray or white matter involvement were reported to achieve good recovery while almost half of the patients with thalamic involvement developed sequelae. The highest mortality rate was among patients with isolated brain stem involvement [2]. Neuropathology is characterized by leptomeningeal mononuclear inflammatory infiltration, and perivascular cuffing, and occasional perivascular demyelination [30]. Current data suggest that EBV-induced CNS pathology is immune mediated.

### Human herpesvirus 6

The two HHV-6 variants, HHV-6A and -B, have recently been re-classified as separate HHVs based on differences in epidemiology, cell tropism and disease association [98]. Whereas no disease has been clearly linked to HHV-6A, HHV-6B can lead to exanthema subitum which has an incubation period of 1–2 weeks. Compared to the other lymphotropic HHVs, HHV6 infection also exhibits neurotropic characteristics. Neurological complications of primary HHV6 infection, mainly HHV-6B, in childhood include seizures, hemiplegia, meningoencephalitis and residual encephalopathy of which outcomes remain uncertain [4]. Poor prognosis has been observed in children with exanthema subitum-associated encephalitis and febrile status epilepticus. Incidentally HHV-6 reactivation in adult transplant patients, mainly bone marrow transplantation involving HHV-6B, may be the causative agent of myelitis, meningitis and post-transplant acute limbic encephalitis [8]. Human neuropathology consists of perivascular mononuclear cell cuffs located in hippocampus (limbic encephalitis), frontal and insular cortices associated with hemorrhages. In the temporal cortex, marked neuronal loss with gliosis and microglial nodules have been described. There are neither viral inclusions, inflammatory demyelination, parenchymatous necrosis nor meningeal inflammation. Immunochemically, HHV-6 antigen is located in nuclei of hippocampal neurons and in astrocytes [37].

### *Orthomyxoviridae*

Neurological complications associated with human orthomyxovirus infections are most commonly associated with Influenza A viruses which are enveloped segmented negative-strand RNA viruses.

### Influenza A viruses

Influenza A viruses are subtyped based on their surface glycoproteins hemagglutinin (HA) and neuraminidase (NA). Many host species have their own strains (e.g., waterbirds, humans, pigs, horses, dogs and bats), although cross-species transmission occurs frequently. Humans can be infected with seasonal, pandemic and zoonotic influenza A viruses. Seasonal influenza A viruses (H3N2 and H1N1) circulate within the population, causing yearly epidemics. Pandemic influenza viruses are the result of cross-species transmission, after which they adapt to humans and spread worldwide. Four pandemics have occurred in the last century, the 1918 H1N1 ‘Spanish flu’, the 1957 H2N2 ‘Asian flu’, the 1968 ‘Hong Kong flu’ and the 2009 H1N1 ‘Mexican flu’ or ‘swine flu’. Zoonotic influenza virus infections in humans are the result of interspecies transmission, without subsequent efficient transmission among humans. The best known examples are avian influenza H7N9 virus and highly pathogenic avian influenza (HPAI) H5N1 virus, which intermittently are transmitted from their poultry reservoirs to humans.

The most common extra-respiratory complication of influenza is the development of CNS disease [71]. Influenza virus infections have been linked to a wide array of neurological diseases, beginning with the 1918 ‘Spanish flu’ pandemic, which was associated with an outbreak of encephalitis lethargica and subsequently post-encephalitis parkinsonism years later [103]. Acute CNS diseases include febrile seizures, acute onset brain dysfunction, meningitis, encephalitis and encephalopathies. In addition, influenza viruses have been linked to the development of Guillan Barré syndrome, Kleine Levin syndrome and transfer myelitis [46, 127]. Besides acute CNS manifestations, influenza viruses have also been associated with neurodegenerative diseases [32]. Interestingly some strains are more frequently associated with CNS disease than others. Recent examples are the 2009 H1N1 pandemic virus and HPAI H5N1 virus [26, 46].

Maternal influenza has been associated with schizophrenia and bipolar disorder (BD) in the offspring. Early studies of prenatal influenza and schizophrenia were ecologic, associating influenza epidemics in populations to patients who would have been in utero during these epidemics [18]. However, these findings were not consistent in all studies. Consequently, studies with greater methodologic rigor were initiated. Unlike prior studies, the presence of influenza infection was determined from antibody-based assays of strains circulating in the population during the period of pregnancies in a large population-based birth cohort investigation known as the Child Health and Development Study (CHDS) [17]. A nested case–control study conducted on this cohort demonstrated a threefold increase in risk of schizophrenia when influenza exposure occurred during the first half of pregnancy [17]. For influenza infection during the first trimester, the risk of schizophrenia was increased sevenfold. However, no increased risk of schizophrenia for influenza exposure was observed during the second half of pregnancy, suggesting specificity to early to mid-pregnancy. Additional studies revealed that maternal influenza was associated with a greater than fourfold, statistically significant increased risk of BD [96]. The strongest effect size was found for BD with psychotic features, with an odds ratio of approximately sixfold. These findings were confirmed in similar assays on archived maternal sera, showing that maternal influenza was related to a greater than fivefold increased risk of BD with psychotic features [19]. There was, however, no increase in risk of BD without psychotic features following maternal influenza exposure. While these findings require replication in independent and larger cohorts, this work suggests that maternal influenza may be a risk factor for psychosis rather than for schizophrenia or BD per se.

**Fig. 6:**
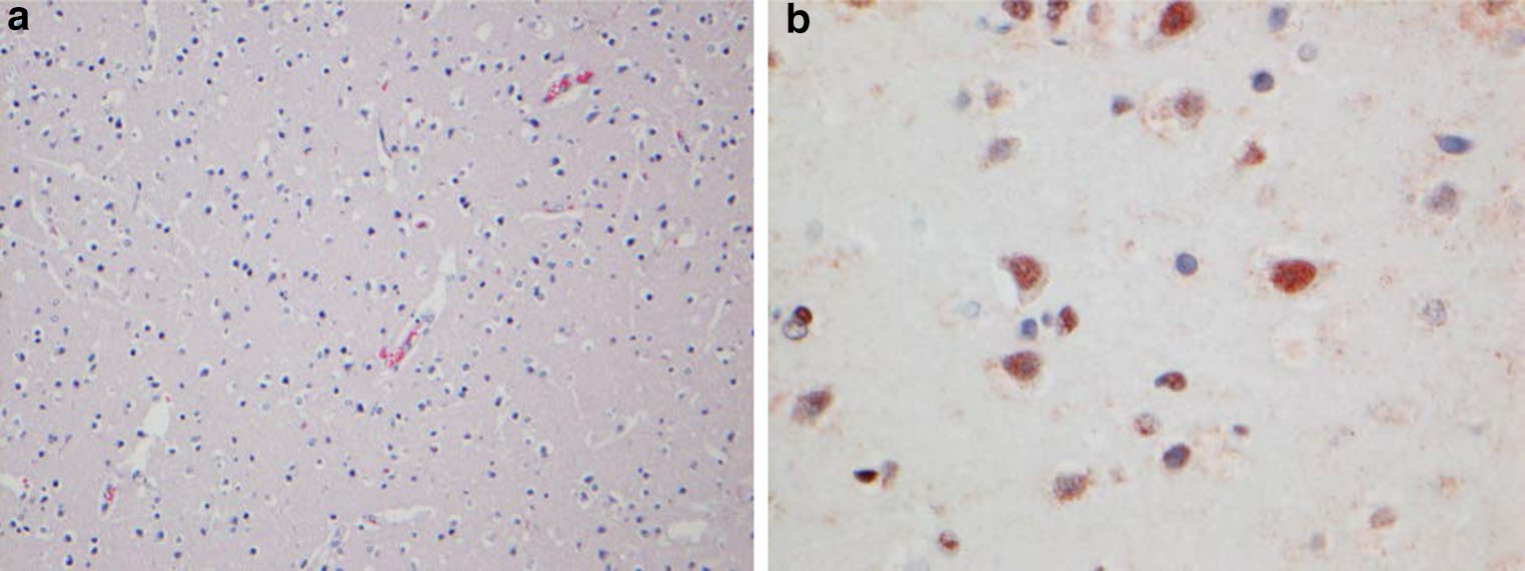
Central nervous system complications of influenza virus infections in humans. Detection of influenza virus antigen in neurons in the olfactory bulb of an H3N2 virus-infected immunocompromised child. **a** Hematoxylin and eosin-stained tissue section showing a lack of cellular infiltrates. **b** Immunohistochemcial detection of influenza virus antigen in neurons using an anti-nucleoprotein monoclonal antibody

The pathogenesis of influenza virus-induced CNS disease in humans is largely unknown. Influenza virus-associated CNS disease could be an indirect effect of the infection, due to systemic cytokines, a direct effect of virus entry into the CNS, or a combination of both [71]. Several studies have detected seasonal or pandemic influenza virus RNA or virus antigen within the CSF or cadaveric CNS tissues (Fig. 6) [111, 118, 132]. In addition, HPAI H5N1 virus has been detected in cadaveric CNS tissues and CSF [26, 70]. The few studies that describe the histopathology of fatal influenza virus-associated CNS disease describe a diffuse cerebral congestion with edema, with an absence of infiltrating inflammatory cells (reviewed by [71]). CNS disease can also be associated with acute sub-arachnoid hemorrhage [118]. Only a few patients will develop acute necrotizing encephalopathy (ANE). This disease is characterized by its fulminant and monophasic course, with multifocal brain lesions bilaterally predominantly in the thalamus, but also in brainstem, periventricular white matter, and cerebellar medulla, often associated with brain oedema [127].

The route of entry into the CNS is not completely understood, but recent data indicate that influenza viruses may enter the CNS directly from the nasal cavity via the olfactory nerve in humans [132]. Virus entry into the CNS via the olfactory nerve might also explain the case reports in which viruses were detected within the nasal cavity and the CNS, without evidence for initial severe LRT disease [26, 118]. With regard to psychiatric disorders from prenatal influenza exposure, there are no studies in humans that have examined neuropathology at the in situ level. The pathomechanisms involved in the development of psychiatric disorders remain enigmatic, but systemic cytokines are believed to be at least partially involved because studies of maternal immune activation in rodents have indicated similar behavioral and electrophysiological effects (for review, see [18]).

### *Paramyxoviridae*

The family *Paramyxoviridae* is comprised of a broad group of enveloped single-stranded negative-sensed RNA viruses of animals and humans. In recent years, our understanding of the true diversity of paramyxoviruses has undergone a renaissance with the discovery of novel paramyxovirus sequences in bats, rodents and birds [33]. However, the zoonotic potential of the viruses from which these sequences were derived is currently unknown. Only a small number of the known paramyxoviruses have been linked to the development of neurological disease in humans. Measles virus (MV), mumps virus (MuV), Hendra virus (HeV) and Nipah virus (NiV) virus are all capable of spreading to the human central nervous system (CNS) during systemic infection. This results in a wide spectrum of disease manifestations and clinical outcomes ranging from transient mild encephalitis or acute fatal encephalitis to long-term infections with severe neurological sequelae.

### Measles virus

Measles virus is the prototype member of the genus morbillivirus and remains a leading cause of morbidity and mortality in the developing world with an estimated 122,000 deaths in 2012 [99]. Clinical signs of MV infection such as fever and a maculopapular rash are visible following an incubation period of approximately 14 days. The pathogenesis of measles is typically characterized by severe immunosuppression and a pronounced epitheliotropism during later disease stages. The propensity of morbilliviruses such as measles virus (MV) or canine distemper virus (CDV) to spread to the central nervous system (CNS) of a susceptible host has been a long-recognized complication of the systemic acute viral infection.

**Fig. 7:**
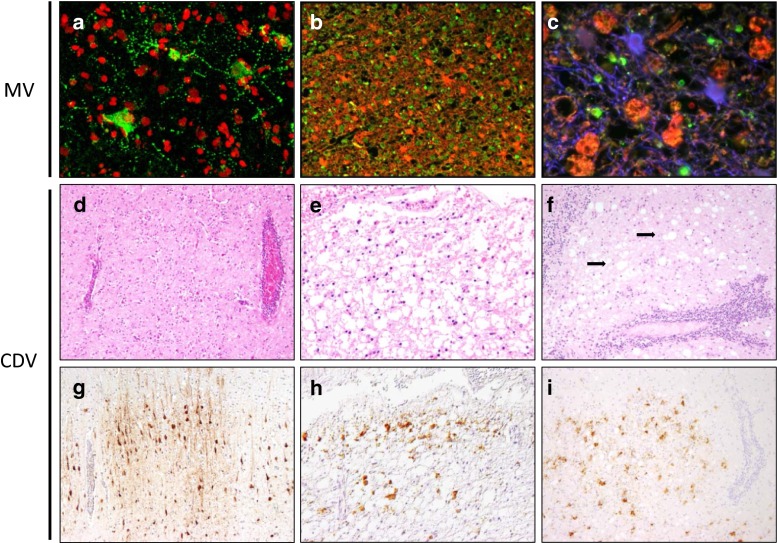
Morbillivirus infection of the CNS. Immunocytochemical detection of measles virus (MV) antigen using anti-nucleoprotein monoclonal antibody (**a**) or SSPE serum (**b**, **c**) in human brain sections from subacute sclerosing panencephalitis (SSPE) patients. **d**, **e** Hematoxylin and eosin-stained brain tissue sections from canine distemper virus (CDV)-infected dogs. **g**–**i** Immunohistochemical detection of CDV-infected cells in dog brain tissue sections. **a** MV antigen (*green*) is present in neurons and associated processes in the occipital lobe of an SSPE case. **b** MV antigen (*green*) is restricted to oligodendrocytes and is not present in GFAP-positive astrocytes (*red*) in SSPE white matter. **c** MV-positive oligodendrocytes in close proximity to CD68-positive macrophages and GFAP-positive astrocytes (*blue*) in white matter from an SSPE case. **d** Cerebrum of a dog with CDV-induced polioencephalitis with perivascular lymphocytic cuffs and diffuse gliosis. **e** Acute leukoencephalitis with severe demyelination in the medulla of a CDV-infected dog. **f** Chronic leukoencephalitis with severe demyelination (*arrows*) and perivascular lymphocytic cuffs is evident in the cerebellum of a CDV-infected dog. **g** Cerebrum of the same dog shown in *panel* **d** with CDV antigen evident in neurons and neuronal processes. **h** Immunolabelling of CDV antigen in astrocytes and gitter cells in the medulla of the same dog shown in *panel* **e**. **i** Cerebellum of the same dog shown in *panel* **f** with CDV antigen present in astrocytes and gitter cells. **a**–**c** Images courtesy of Dr. Stephen McQuaid (Belfast Health and Social Care Trust, Northern Ireland)

**Fig. 8:**
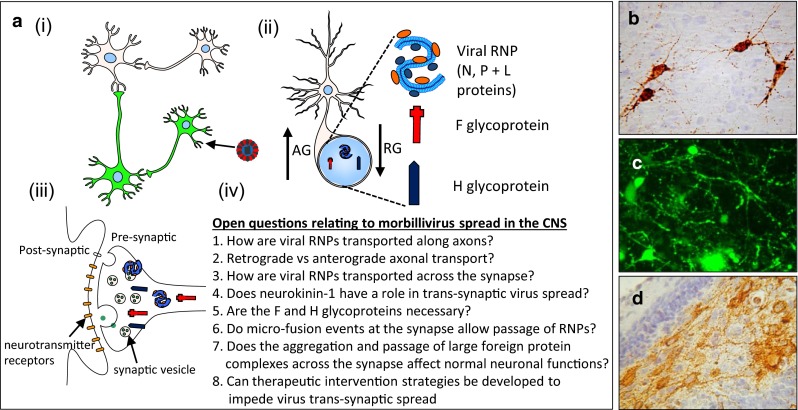
Measles virus (MV) spread in the CNS. **a** Schematic diagrams illustrating the trans-synaptic spread of MV. (*i*) Neurons are initially infected by MV via an uncharacterized route of infection. Following virus entry MV is able to spread to connected uninfected neurons. (*ii*) Viral ribonucleoprotein (RNP) complexes consisting of viral genome encapsidated by nucleoprotein with associated phosphoprotein and polymerase are transported together with the fusion (*F*) and hemagluttinin (*H*) glycoproteins along axons to the synaptic termini. Axonal transport of viral RNPs is known to occur via both anterograde (*AG*) or retrograde (*RG*) axonal transport. (*iii*) Aggregation of viral RNPs and viral glycoproteins at the pre-synaptic terminal. (*iv*) A number of questions relating to the mechanism and consequences of MV spread in the CNS remain to be addressed. (**b**–**d**) MV transneuronal spread in human brain (**b**) and animal models (**c**, **d**). **b** Interconnected MV-infected neurons are present in brain sections from an SSPE patient (**b**), a rMV-infected Ifnarko-CD46Ge mouse (**c**). **d** A number of extended neuronal processes (*arrows*) connect infected neurons in the polymorph cell layer of the hippocampus of a C57/BL/6 mouse infected with the rodent-adapted MV^CAM/RB^ strain. **a** Plate was composed using ©Motifolio.com Biomedical PowerPoint Toolkit Suite; **b** image courtesy of Dr. Stephen McQuaid (Belfast Health and Social Care Trust, Northern Ireland); **c** adapted from [76]; **d** adapted from [75]

Acute demyelinating encephalomyelitis (ADME) typically occurs in 1:1000 measles patients. Few studies have definitively linked MV infection to this condition. Instead, ADME is considered an autoimmune disease in which an acute virus infection leads to inflammation of the brain with concomitant myelin damage. Acute lesions are disseminated throughout the CNS and are characterised by perivenous infiltration and demyelination [82]. In most cases, patients recover with no complications but in <5 % cases disease is fatal. Measles inclusion body encephalitis (MIBE) occurs in immunosuppressed individuals 3–6 months following an acute MV infection. These cases are typically associated with patients who are immunosuppressed due to HIV infection or who take immunosuppressive drugs as a result of organ transplantation [3, 52]. Subacute sclerosing panencephalitis (SSPE) occurs on average 4–10 years following acute MV infection. The frequency of SSPE following MV infection <5 years of age is estimated to be approximately 1:1700 to 1:3300 [112]. The development of clinical signs of encephalitis are commonly preceded or concomitant with ocular disturbances [39], but it is currently unknown if this occurs as a direct result of MV infection or indirectly via immunopathology. The neuropathology of MIBE and SSPE are similar and both are characterised by extensive infection of neurons and oligodendrocytes throughout the brain including the frontal cortex, occipital cortex, basal ganglia, thalamus pons, medulla and parietal cortex (Fig. 7a–c) [80]. Expression of MV antigen throughout neuronal processes is also commonly observed in the brain (Fig. 8b–d) [6]. Extensive perivascular infiltrates occur throughout the brain and in the later stages of the infection, nerve cell degeneration and gliosis are commonly observed [86]. CDV has served in useful model systems in which morbillivirus CNS spread during natural and experimental infections could be readily assessed (Fig. 7d–i). Analysis of MV sequences obtained from deceased MIBE and SSPE patients showed extensive mutations especially in genomic regions encoding determinants of virus assembly and/or cell-to-cell spread such as the matrix (M) protein or the cytoplasmic tail of the fusion (F) glycoprotein. It is assumed that these mutations facilitate the long-term persistence and spread of the virus within the CNS.

Specific mechanisms of MV spread within the CNS are unknown but detailed pathological and ultrastructural studies of SSPE brain tissue have indicated that the mechanism of MV spread in the CNS is profoundly different from that observed in non-neural cells. MV infection of the CNS is characterised by a lack of virus budding and an absence of giant cell formation. This has led to the suggestion that measles virus spread between neural cells occurs through lateral cell–cell contacts in the absence of appreciable levels of cell-to-cell fusion [6]. In spite of major advances in our understanding of how MV enters the body, spreads systemically and is released into the respiratory tract, our understanding of how wild-type non-tissue culture adapted MV transverses the synapse has stalled beyond observations made in in vivo model systems. While morbilliviruses clearly can spread trans-synaptically within the brain, many questions remain as to the underlying mechanism and consequences of this for normal neuronal functioning (Fig. 8).

### Mumps virus

Mumps virus (MuV) is a member of the genus *Rubulavirus* and causes a disease that was first accurately described by Hippocrates in the first book of the *Epidemics*. Alongside the more common complication orchitis, the neurological complication of mumps was first noted by Robert Hamilton in 1758. In spite of the ubiquitous nature of MuV as a common childhood disease in the pre-vaccine era, surprisingly little is known about the pathogenesis of mumps. MuV infects a susceptible host via the respiratory tract and then spreads to local lymph nodes where amplification of the virus results in systemic virus spread. Following an incubation period of 7–21 days, the classical signs of mumps develop, such as bilateral swelling of the parotid glands (parotitis). MuV also infect the CNS and although estimates vary, it has been suggested that this can occur in up to 30 % of cases with approximately 1:6000 cases complicated by more severe MuV encephalitis [108]. In the pre-vaccine era MuV was the leading cause of aseptic meningitis which was typically mild, characterised by pleocytosis of the CSF and resolved with few complications. This CNS complication commonly occurred in the absence of classical signs of MuV infection. In one large study, parotitis was only detected in 51 of 131 cases (37 %) of mumps meningitis [63]. The more severe mumps encephalitis can occur at the peak of acute infection (during parotitis) or up to 23 days later with death occurring in up to 20 % of these cases while 33 % of survivors show evidence of prolonged neurological sequela [108]. Pathologically, mumps encephalitis resembles ADME and is characterised by extensive perivascular demyelination in the white matter of the cerebral and cerebellar hemispheres, basal ganglia, midbrain, pons, medulla and spinal cord with areas of demyelination associated with the perivascular infiltration of immune cells, microglia and gliosis [31].

### Henipaviruses

Nipah virus (NiV) and Hendra virus (HeV) virus, both members of the genus *Henipavirus* are zoonotic viruses which are capable of inducing severe neurological disease in humans. This occurs following virus spread via intermediate animal hosts from flying foxes of the genus Pteropus which serve as the reservoir host for these viruses. HeV was first detected in Queensland in 1994 following a disease outbreak in 21 racehorses and two humans and was rapidly identified as a novel paramyxovirus [114]. NiV was identified as the etiological agent responsible for a large outbreak of encephalitis among 229 pig farmers in Malaysia in 1998 [20]. Human NiV infections occurred as a direct result of virus spread from infected pigs. Subsequent smaller outbreaks have occurred in India and in more recent years annual cases of NiV infection in Bangladesh have been documented with infections occurring as a result of the consumption of NiV-contaminated date palm sap. In one case of HeV infection and twenty cases of NiV infection, relapsing encephalitis was observed a number of months after the initial acute infection resulting in severe neurological sequela with many parallels to the MV-induced neurological complication SSPE [6]. Prodromal signs of henipavirus infection are general and include fever, lethargy and headache and occur following an incubation period of 5–14 days. This rapidly progresses to neurological signs of infection including limb weakness and segmental myoclonus. The neuropathology of henipavirus infection in humans is characterised by virus infection of neurons and disseminated small vessel vasculopathy which is particularly severe in the CNS. Virus infection of cerebral endothelial cells results in giant cell formation which may contribute to vasculitis and intravascular thrombosis in cerebral blood vessels [146, 147]. Such pathology is commonly associated with necrotic inflammatory lesions throughout the brain. In cases of relapsing henipavirus encephalitis, neuropathology involves extensive parenchymal necrosis and oedema, gliosis and prominent infiltrates of macrophages and lymphocytes often with associated meningitis with virus-positive neurons and ependymal cells readily detected [145, 147]. However, in contrast to the acute CNS infection, vasculitis is not observed. Many questions relating to this late complication of henipavirus infection remain to be elucidated, especially any similarities to late complications of MV infection such as SSPE.

### *Picornaviridae*

The family *Picornaviridae* is one of the largest virus families consisting of small, non-enveloped single-stranded RNA viruses. The family is classified into 29 genera including many human pathogens such as poliovirus (PV) and the non-polio enteroviruses (EVs) of the *Enterovirus* genus, human parechovirus (HPeV) and hepatitis A virus. Animal pathogens include foot-and-mouth disease virus and Theiler’s murine encephalomyelitis virus. Clinical symptoms caused by picornaviruses in humans vary greatly, ranging from the common cold to life-threatening infections such as encephalitis and myocarditis. Hereafter, picornaviruses that can cause neurological disease in humans will be discussed. CNS infections are most often caused by EVs and are estimated to cause 10–15 million infections in the United States with at least 30,000–50,000 hospitalizations per year, that are mainly due to aseptic meningitis [95]. Based on molecular and serological characteristics the EVs are classified into species EV-A, containing EV71 and several Coxsackie A viruses (CV-A), species EV-B including coxsackie B viruses (CVB) 1–6 and all echoviruses, EV-C with the polioviruses (PVs) 1–3 and several CVAs, and EV-D containing EV-68 [129]. Following EVs, the second most frequent viral cause of CNS disease is HPeV [143], which belongs to the *Parechovirus* genus. The HPeV species are only found in primates and now consists of 16 types [12]. The disease spectrum is similar to that of EV infections, although HPeV infection is almost exclusively seen in children.

### Enteroviruses

Enterovirus infections of the brain are caused by a variety of EV genotypes and occur most often in children under 10 years of age. EVs account for more than 90 % of viral meningitis cases, while encephalitis only occurs in 3 % of neurological EV infections [107, 129]. Meningitis due to EVs is most frequently caused by echoviruses and CVB. Furthermore, CNS infections have been associated with AFP, disseminating myelitis and transverse myelitis [105]. The most global public health threat was attributed to PV causing AFP (poliomyelitis) often leading to permanent disabilities. Neuropathology of EV infection comprises lympho-histiocytic leptomeningitis with edema and predominantly perivascular lymphocytic cuffs in the neuroparenchyma with occasional neutrophils. In both, encephalitis or encephalomyelitis, gliosis, microglial nodules, neuronophagia, neutrophilic infiltrates and necrosis may occur. Viral antigen is expressed in neurons, particularly of the brain stem and neuronal processes [88]. Neuropathology varies considerably depending on the stage of the disease. In acute cases, hemorrhagic necrosis may be found grossly in the anterior horns of the spinal cord. In chronic cases, there is atrophy of the anterior spinal nerve roots and a reduced size of the corresponding anterior gray horn. Histological changes in the acute phase comprise diffuse lymphocytic spinal leptomeningitis and lymphocytic myelitis of the anterior horns, occasionally associated with infiltration of neutrophils and occurrence of inclusion bodies in anterior horn cells. Subsequently, necrosis of motor neurons occurs with activation of microglial cells and development of microglial nodules. In fatal cases, these necrotic, reactive and inflammatory changes may obscure the normal architecture of the spinal cord. In chronic cases, the inflammatory infiltration decreases, neurophagia and microglial hyperplasia represents the most prominent finding. Additionally, plasma cellular infiltration occurs. In very late stages of the disease, loss of neurons in the anterior horn with focally extensive gliosis, loss of myelinated fibers in the affected region and atrophy of the corresponding anterior spinal root nerves are seen [74]. Since the global polio vaccination roll-out in the 1950s and 1960s, poliomyelitis incidence is virtually eliminated with the exception of circulating strains in Pakistan, Afghanistan and Nigeria [138]. Since then world surveillance studies as recommended by the World Health Organisation allowed for accurate monitoring of other causes of AFP.

Picornaviruses are transmitted via the oral–fecal route. Following transmission, the sites of primary replication are considered the respiratory and gastrointestinal tract. From there, the virus spreads via the blood to a variety of target organs. EVs can infect many different target cells including epithelial cells, neurons and cardiomyocytes [88]. There is increasing evidence that EVs are capable of infecting the CNS through distinct pathways. While enterovirus 71 most likely spreads to the brain through the motor but not peripheral sensory or autonomic pathways [144], PV can enter the CNS either directly from the blood or by retrograde axonal transport when PV enters the neuromuscular junction [101].

The local immune response against picornavirus CNS infections is largely unknown and is mainly deducted from immunological evaluations in EV infection. Undoubtedly an adequate humoral response with release of neutralizing antibodies is crucial as a defence mechanism. Indeed, patients with antibody deficiencies, such as X-linked agammaglobulinemia, are at increased risk for chronic enteroviral meningoencephalitis and long-term neurological symptoms [48]. Lack of specific maternal EV antibodies in neonates is a risk factor for the development of severe illness further emphasizing the need for a humoral immune response in EV infection [51]. In contrast to EV infections, no data are available that favor a protective role of neutralizing antibodies for HPeV CNS infections. Toll-like receptor (TLR)-mediated cytokine expressions are increasingly being recognized as important pathogenic or protective mechanisms. For example, EVD68 inhibits TLR3-mediated immunity that triggers interferon-beta(IFN-β) expression and activation [149].

Other EVs have emerged more recently. From the late 1990s, EV71 has caused massive outbreaks in the Asian Pacific region associated with brain stem encephalitis causing hundreds of deaths mainly in children [122]. In addition, EV71 and CV-A were linked to neurological disease such as Guillain–Barré syndrome and myelitis (including transverse myelitis). EVD68 was first isolated from children with lower respiratory tract infections in 1967, but as the incidence of AFP also occurred during EVD68 outbreaks [48], the association with neurologic disease was further investigated. In clinical practice, neurological symptoms such as vomiting, irritability, and nuchal rigidity in young children should trigger clinicians to focus on diagnosing EV infections. Beside CNS-specific symptoms, EV-induced clinical symptoms are aspecific including poor feeding, general malaise and low-grade fever. Some EVs, however, have strong unique clinical associations. For example, EV71 has been associated with neurogenic pulmonary edema [88].

### Human Parechoviruses

Human Parechovirus infections are usually associated with (upper) respiratory tract and gastrointestinal infections in young children, although incidentally severe neurologic disease such as flaccid paralysis, meningitis, encephalitis and encephalomyelitis has been reported. The clinical picture is clearly different from the other HPeV infections. HPeV3 infection in adults is often asymptomatic or elicits minor disease such as myalgia [83], whereas severe HPeV3 disease in immunocompromised adults is increasingly acknowledged [78]. Children infected with HPeV3 are usually younger than 3 months of age and often present with fever, feeding problems and irritability as signs of CNS involvement [12]. In neonatal encephalitis, HPeV3 infection shows specific white matter involvement extending into the subcortical white matter involving entire tracts of fibers. A recent study was able to demonstrate HPeV3 infection of meningothelial and vascular smooth muscle cells in two neonates with fatal leukoencephalopathy, suggesting that dysfunction of infected blood vasculature may have primarily caused the periventricular white matter lesions in these patients [13]. Severity of white matter lesions is predictive of neurodevelopmental outcomes in these children [136]. HPeV1 uses members of the integrin family as cellular receptor(s) [64]; however, the HPeV3 receptor is not yet identified and will likely be different. Differential receptor expression on target cells further defines tissue tropism. The transport of the virus from its primary replication sites to the brain has not been fully elucidated, and may be realized by crossing the blood–brain barrier or via retrograde transaxonal transport [105, 129]. The pathogenicity and tropism of HPeVs has not been yet been fully established yet.

### *Rhabdoviridae*

The family *Rhabdoviridae* comprises 11 genera, of which viruses within the genus Lyssavirus and Vesiculovirus are of medical and veterinary importance. The genus Lyssavirus contains seven genotypes: rabies virus (RABV; genotype 1), Lagos bat virus (genotype 2), Mokola virus (genotype 3), Duvenhage virus (genotype 4), European Bat lyssaviruses type 1 and 2 (genotypes 5 and 6), and Australian bat lyssavirus (genotype 7). RABV is prevalent throughout the world with only few countries being rabies free. The virus infects mammalian species, most frequently carnivores and bats. Genotypes 2 through 7 are geographically restricted and have bat reservoirs. The genus Vesiculovirus contains the vesicular stomatitis virus (VSV), an arthropod-borne virus that infects primarily rodents, swine, horses, and cattle. Humans only experience mild symptoms upon VSV infection. Currently there is no effective treatment for clinical rabies and a better understanding of the pathogenesis is needed to develop better post-exposure treatment regimens and curative treatments for this invariably lethal disease.

### Rabies virus

Rabies is a neurological disease characterized by a fatal encephalitis, caused by RABV or one of the other lyssaviruses, which are usually transmitted through a bite or scratch of infected carnivores (terrestrial rabies) or bats (bat rabies). Rabies presents as either furious or paralytic rabies (67 and 33 %, respectively). Approximately 55,000 to 60,000 people are reported to die annually as a result of rabies. Clinical manifestations of terrestrial and bat rabies are different [130]. This difference may be associated with differences between strains of RABV from terrestrial carnivores and bats, as well as the route of viral spread. Patients infected with RABV typically go through different stages of disease: the incubation, prodromal, neurological, and comatose stages and finally death. The incubation time of rabies is on average 2 months, but can vary from weeks to up to 1 year. In the prodromal phase, patients may experience mild and non-specific symptoms such as fever and gastrointestinal discomfort. In the neurological phase, furious rabies patients may develop paresthesia at the site of exposure, anxiety or agitation, dysautonomia (autonomic dysfunction) including inspiratory spasms, hypersalivation hydrophobia, sometimes in combination with aerophobia and fluctuating mental state. Later in the disease, patients may develop altered levels of consciousness, before they eventually enter into the comatose stage. Patients with furious rabies who enter the comatose stage may develop flaccid limb weakness, which could be misinterpreted as paralytic rabies. The last stage of the disease is a painful and excruciating death. The paralytic form of rabies is rather characterized by progressive weakness and paralysis, with absence of many of the symptoms seen in the neurologic phase of furious rabies. Paralytic rabies is often misdiagnosed as Guillain–Barré syndrome (GBS), autoimmune disease or stroke. In general, weakness is the initial manifestation in paralytic rabies and GBS. Paralytic rabies is more often seen in bat rabies and especially in patients who received post-exposure vaccination [44]. The muscle weakness observed in paralytic rabies is likely caused by peripheral nerve dysfunction and/or the involvement of anterior horn cells or of motor nerve fibers. Bat-associated RABV infection usually presents with myoclonus, hemichorea and symptoms related to a one-side deficiency of the sympathetic trunk activity, such as a weak, droopy eyelid, constricted pupil, decreased sweating and in some cases an inset eyeball [54, 130]. The presence of myoedema and urinary incontinence in patients differentiate paralytic rabies from GBS.

It is generally believed that low titer virus exposure will first lead to one or more rounds of replication in muscle cells before motor neurons at the neuromuscular junctions are infected. In contrast, exposure to a high virus titer, leads to direct infection of motor neurons whereas sensory neurons are probably not infected. Nevertheless, several cases of rabies following skin lesions have been reported [47, 133], although it is not clear whether sensory neurons were infected in those cases. It is possible that different genotype 1 lyssaviruses have differential tropism for sensory neurons and epidermal cells [85]. Following infection through a bite of an infected animal, the virus infects motor neurons and reaches the spinal cord by retrograde transaxonal transport, where it infects interneurons and neurons of the dorsal root ganglia (DRG) innervating the bitten extremity. Small DRG neurons that target higher order interneurons in the dorsal horn are subsequently infected [54], eventually leading to infection of the brain stem and the limbic system. Once in the CNS, RABV spreads throughout the brain and migrates back to the periphery probably via the DRG late in the infection process by anterograde transaxonal transport [87]. Late in the disease course the virus can be found in skin, salivary glands, heart, kidney and cornea. However, it is unclear if the virus is only present in the nerve endings or also in the parenchyma of the different organs. Consequently, virus tropism for salivary glands and lacrimal glands remains unexplained, as infectious RABV can be recovered from the saliva, tears and tracheobronchial secretions.

**Fig. 9:**
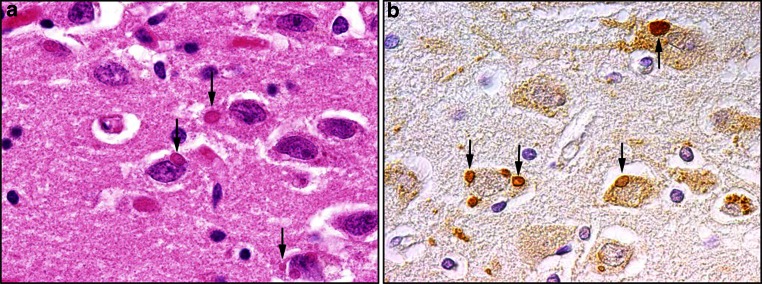
Characterisation of fatal rabies encephalitis in the hippocampus of a 46-year-old human who received a lung transplant from a rabies virus-infected donor. **a** Hematoxylin and eosin staining showing eosinophilic cytoplasmic inclusion bodies (Negri bodies) in the perikaryon of neurons (*arrows*). **b** Immunohistochemical labeling using polyclonal goat anti-rabies antisera of rabies viral antigen in the perikaryon of neurons (*arrows*)

Air-borne infection with RABV or infection via transplantation of infected tissues or organs represents uncommon modes of transmission [125, 140]. A classical event of multiple organ transplantation-related rabies occurred in Germany in 2010. A RABV-infected dog in India bit a 26-year-old female drug user. Unfortunately, the infection was interpreted as toxic psychosis 8 weeks after return to Germany. Her lungs, kidneys, pancreas, liver and cornea were transplanted into six different patients [142]. The 46-year-old female lung recipient died within 7 weeks after showing neurological signs. Histologically, a multifocal mild non-suppurative menigoencephalitis with cytoplasmic eosinophilic inclusion bodies (Negri bodies) in neurons was present in different brain regions (Fig. 9a). Rabies viral antigen was detected by immunohistochemical staining in neurons of various brain regions (Fig. 9b) and the spinal cord. Virus was localized in axons and dendrites, in numerous peripheral nerves of the transplanted lung as well as in nerves of the thyroid gland, kidneys, pancreas, salivary gland and in ganglia of the intestinal wall. Electron microscopy confirmed RABV in the brain. In general, RABV is found in several areas of the brain of patients with furious rabies, including cerebellum, brainstem, the limbic system and the cortex. Systematic studies of RABV distribution in brains of humans who died with furious or paralytic rabies are lacking. There is evidence from infected dogs that viral protein expression in the brain differs between furious and paralytic rabies, in that more antigen is detected in the cerebrum of furious cases, whereas brainstem inflammation is more pronounced in paralytic cases [116].

## Conclusion

Infections with a wide range of viruses from different virus families may cause immediate or delayed neuropathological changes and neurological manifestations in humans and animals worldwide. The ability of a virus to cause an acute or more long-term infection of the CNS is undoubtedly due to a complex milieu of many factors including host genetics, specific interaction with the host immune system, capacity to spread rapidly to immunoprivileged sites such as the brain and unique modalities of cell-to-cell spread within the CNS. Collectively these interactions determine the speed and severity of the disease course. A better understanding of the molecular, epidemiological and biological characteristics of these infections and in particular of the mechanisms that underlie neuropathological or immune-pathological alterations associated with their clinical manifestations are expected to provide tools for the development of more effective intervention strategies and treatment regimens. This will be of critical importance in the event of future novel neurotropic infections caused by zoonotic viruses.

## Electronic supplementary material

Supplementary material 1 (DOCX 93 kb)

## References

[1] Abdel-Wahab KS, El Baz LM, El-Tayeb EM, Omar H, Ossman MA, Yasin W (1978). Rift Valley Fever virus infections in Egypt: pathological and virological findings in man. Trans R Soc Trop Med Hyg.

[2] Abul-Kasim K, Palm L, Maly P, Sundgren PC (2009). The neuroanatomic localization of Epstein–Barr virus encephalitis may be a predictive factor for its clinical outcome: a case report and review of 100 cases in 28 reports. J Child Neurol.

[3] Agamanolis DP, Tan JS, Parker DL (1979). Immunosuppressive measles encephalitis in a patient with a renal transplant. Arch Neurol.

[4] Agut H, Bonnafous P, Gautheret-Dejean A (2015). Laboratory and clinical aspects of human herpesvirus 6 infections. Clin Microbiol Rev.

[5] Alker A (2015). West Nile virus-associated acute flaccid paralysis. BMJ Case Rep.

[6] Allen IV, McQuaid S, McMahon J, Kirk J, McConnell R (1996). The significance of measles virus antigen and genome distribution in the CNS in SSPE for mechanisms of viral spread and demyelination. J Neuropathol Exp Neurol.

[7] Allen R, Taylor SK, Sulkin SE (1970). Studies of arthropod-borne virus infections in Chiroptera. 8. Evidence of natural St. Louis encephalitis virus infection in bats. Am J Trop Med Hyg.

[8] Aoki K, Arima H, Kato A, Hashimoto H, Tabata S, Matsushita A, Ishikawa T (2012). Human herpes virus 6-associated myelitis following allogeneic bone marrow transplantation. Ann Hematol.

[9] Armangue T, Leypoldt F, Málaga I, Raspall-Chaure M, Marti I, Nichter C (2014). Herpes simplex virus encephalitis is a trigger of brain autoimmunity. Ann Neurol.

[10] Arpino C, Curatolo P, Rezza G (2009). Chikungunya and the nervous system: what we do and do not know. Rev Med Virol.

[11] Baldelli F, Ciufolini MG, Francisci D, Marchi A, Venturi G, Fiorentini C (2004). Unusual presentation of life-threatening Toscana virus meningoencephalitis. Clin Infect Dis.

[12] Benschop K, Wildenbeest J, Pajkrt D, Wolthers K (2012). Human Parechoviruses, new players in the pathogenesis of viral meningitis. Meningitis.

[13] Bissel SJ, Auer RN, Chiang CH, Kofler J, Murdoch GH, Nix WA (2015). Human Parechovirus 3 meningitis and fatal leukoencephalopathy. J Neuropathol Exp Neurol.

[14] Borgherini G, Poubeau P, Staikowsky F, Lory M, Le Moullec N, Becquart JP (2007). Outbreak of chikungunya on Reunion Island: early clinical and laboratory features in 157 adult patients. Clin Infect Dis.

[15] Bourg M, Herzog S, Encarnacao JA, Nobach D, Lange-Herbst H, Eickmann M, Herden C (2013). Bicolored white-toothed shrews as reservoir for borna disease virus, Bavaria, Germany. Emerg Infect Dis.

[16] Breuer J, Pacou M, Gauthier A, Brown MM (2014). Herpes zoster as a risk factor for stroke and TIA: a retrospective cohort study in the UK. Neurology.

[17] Brown AS, Begg MD, Gravenstein S, Schaefer CA, Wyatt RJ, Bresnahan M, Babulas VP, Susser ES (2004). Serologic evidence of prenatal influenza in the etiology of schizophrenia. Arch Gen Psychiatry.

[18] Brown AS, Derkits EJ (2010). Prenatal infection and schizophrenia: a review of epidemiologic and translational studies. Am J Psychiatry.

[19] Canetta SE, Bao Y, Co MD, Ennis FA, Cruz J, Terajima M, Shen L, Kellendonk C, Schaefer CA, Brown AS (2014). Serological documentation of maternal influenza exposure and bipolar disorder in adult offspring. Am J Psychiatry.

[20] Centers for Disease C, Prevention (1999). Outbreak of Hendra-like virus—Malaysia and Singapore, 1998–1999. Morb Mortal Wkly Rep.

[21] Cerna F, Mehrad B, Luby JP, Burns D, Fleckenstein JL (1999). St. Louis encephalitis and the substantia nigra: MR imaging evaluation. Am J Neuroradiol.

[22] Cheeran MCJ, Lokensgard JR, Schleiss MR (2009). Neuropathogenesis of congenital cytomegalovirus infection: disease mechanisms and prospects for intervention. Clin Microbiol Rev.

[23] Chen WR, Tesh RB, Rico-Hesse R (1990). Genetic variation of Japanese encephalitis virus in nature. J Gen Virol.

[24] Chow FC, Glaser CA, Sheriff H, Xia D, Messenger S, Whitley R, Venkatesan A (2015). Use of clinical and neuroimaging characteristics to distinguish temporal lobe herpes simplex encephalitis from its mimics. Clin Infect Dis.

[25] Chowers MY, Lang R, Nassar F, Ben-David D, Giladi M, Rubinshtein E (2001). Clinical characteristics of the West Nile fever outbreak, Israel, 2000. Emerg Infect Dis.

[26] de Jong MD, Bach VC, Phan TQ, Vo MH, Tran TT, Nguyen BH (2005). Fatal avian influenza A (H5N1) in a child presenting with diarrhea followed by coma. N Engl J Med.

[27] de la Monte S, Castro F, Bonilla NJ, Gaskin de Urdaneta A, Hutchins GM (1985). The systemic pathology of Venezuelan equine encephalitis virus infection in humans. Am J Trop Med Hyg.

[28] Deresiewicz RL, Thaler SJ, Hsu L, Zamani AA (1997). Clinical and neuroradiographic manifestations of eastern equine encephalitis. N Engl J Med.

[29] Dionisio D, Valassina M, Ciufolini MG, Vivarelli A, Esperti F, Cusi MG (2001). Encephalitis without meningitis due to sandfly fever virus serotype toscana. Clin Infect Dis.

[30] Doja A, Bitnun A, Jones EL, Richardson S, Tellier R, Petric M, Heurter H, Macgregor D (2006). Pediatric Epstein–Barr virus-associated encephalitis: 10-year review. J Child Neurol.

[31] Donohue WL, Playfair FD, Whitaker L (1955). Mumps encephalitis; pathology and pathogenesis. J Pediatr.

[32] Doty RL (2008). The olfactory vector hypothesis of neurodegenerative disease: is it viable?. Ann Neurol.

[33] Drexler JF, Corman VM, Muller MA, Maganga GD, Vallo P, Binger T (2012). Bats host major mammalian paramyxoviruses. Nat Commun.

[34] Durrwald R, Kolodziejek J, Herzog S, Nowotny N (2007). Meta-analysis of putative human bornavirus sequences fails to provide evidence implicating Borna disease virus in mental illness. Rev Med Virol.

[35] Dutta K, Kumawat KL, Nazmi A, Mishra MK, Basu A (2010). Minocycline differentially modulates viral infection and persistence in an experimental model of Japanese encephalitis. J Neuroimmune Pharmacol.

[36] Eberhardt O, Kuker W, Dichgans J, Weller M (2004). HSV-2 sacral radiculitis (Elsberg syndrome). Neurology.

[37] Forest F, Duband S, Pillet S, Stachowicz ML, Cornillon J, Dumollard JM, Peoc’h M (2011). Lethal human herpesvirus-6 encephalitis after cord blood transplant. Transpl Infect Dis.

[38] Fujino K, Horie M, Honda T, Merriman DK, Tomonaga K (2014). Inhibition of Borna disease virus replication by an endogenous bornavirus-like element in the ground squirrel genome. Proc Natl Acad Sci U S A.

[39] Gagnon A, Bouchard RW (2003). Fulminating adult-onset subacute sclerosing panencephalitis in a 49-year-old man. Arch Neurol.

[40] Ganesan K, Diwan A, Shankar SK, Desai SB, Sainani GS, Katrak SM (2008). Chikungunya encephalomyeloradiculitis: report of 2 cases with neuroimaging and 1 case with autopsy findings. Am J Neuroradiol.

[41] Garen PD, Tsai TF, Powers JM (1999). Human eastern equine encephalitis: immunohistochemistry and ultrastructure. Mod Pathol.

[42] Gelpi E, Preusser M, Garzuly F, Holzmann H, Heinz FX, Budka H (2005). Visualization of Central European tick-borne encephalitis infection in fatal human cases. J Neuropathol Exp Neurol.

[43] Gerardin P, Barau G, Michault A, Bintner M, Randrianaivo H, Choker G (2008). Multidisciplinary prospective study of mother-to-child chikungunya virus infections on the island of La Reunion. PLoS Med.

[44] Ghosh JB, Roy M, Lahiri K, Bala AK, Roy M (2009). Acute flaccid paralysis due to rabies. J Pediatr Neurosci.

[45] Gilden D, Nagel MA, Cohrs RJ (2014). Varicella-zoster. Neurovirology.

[46] Glaser CA, Winter K, DuBray K, Harriman K, Uyeki TM, Sejvar J, Gilliam S, Louie JK (2012). A population-based study of neurologic manifestations of severe influenza A(H1N1)pdm09 in California. Clin Infect Dis.

[47] Gowda VK, Basavaraja GV, Reddy H, Ramaswamy P (2014). Paralytic rabies following cat scratch and intra-dermal anti-rabies vaccination. J Pediatr Neurosci.

[48] Greninger AL, Naccache SN, Messacar K, Clayton A, Yu G, Somasekar S, Federman S (2015). A novel outbreak enterovirus D68 strain associated with acute flaccid myelitis cases in the USA (2012–14): a retrospective cohort study. Lancet Infect Dis.

[49] Griffiths MJ, Lemon JV, Rayamajhi A, Poudel P, Shrestha P, Srivastav V (2013). The functional, social and economic impact of acute encephalitis syndrome in Nepal—a longitudinal follow-up study. PLoS Negl Trop Dis.

[50] Haddow AD, Bixler D, Odoi A (2011). The spatial epidemiology and clinical features of reported cases of La Crosse virus infection in West Virginia from 2003 to 2007. BMC Infect Dis.

[51] Halliday E (2003). Enteroviral infections in primary immunodeficiency (PID): a survey of morbidity and mortality. J Infect.

[52] Hardie DR, Albertyn C, Heckmann JM, Smuts HE (2013). Molecular characterisation of virus in the brains of patients with measles inclusion body encephalitis (MIBE). Virol J.

[53] Hartman AL, Powell DS, Bethel LM, Caroline AL, Schmid RJ, Oury T, Reed DS (2014). Aerosolized rift valley fever virus causes fatal encephalitis in african green monkeys and common marmosets. J Virol.

[54] Hemachudha T, Ugolini G, Wacharapluesadee S, Sungkarat W, Shuangshoti S, Laothamatas J (2013). Human rabies: neuropathogenesis, diagnosis, and management. Lancet Neurol.

[55] Herden C, Briese T, Lipkin WI, Richt JA, Knipe DM, Howley PM (2013). Bornaviridae. Fields virology.

[56] Hilbe M, Herrsche R, Kolodziejek J, Nowotny N, Zlinszky K, Ehrensperger F (2006). Shrews as reservoir hosts of borna disease virus. Emerg Infect Dis.

[57] Hoffmann B, Tappe D, Hoper D, Herden C, Boldt A, Mawrin C, Niederstrasser O, Muller T, Jenckel M, van der Grinten E (2015). A variegated squirrel bornavirus associated with fatal human encephalitis. N Engl J Med.

[58] Honkavuori KS, Shivaprasad HL, Williams BL, Quan PL, Hornig M, Street C (2008). Novel borna virus in psittacine birds with proventricular dilatation disease. Emerg Infect Dis.

[59] Horie M, Honda T, Suzuki Y, Kobayashi Y, Daito T, Oshida T, Ikuta K, Jern P, Gojobori T, Coffin JM (2010). Endogenous non-retroviral RNA virus elements in mammalian genomes. Nature.

[60] Hornig M, Briese T, Licinio J, Khabbaz RF, Altshuler LL, Potkin SG (2012). Absence of evidence for bornavirus infection in schizophrenia, bipolar disorder and major depressive disorder. Mol Psychiatry.

[61] Iwamasa T, Yoshitake H, Sakuda H, Kamada Y, Miyazato M, Utsumi Y, Nakamura A (1991). Acute ascending necrotizing myelitis in Okinawa caused by herpes simplex virus type 2. Virchows Arch A Pathol Anat Histopathol.

[62] Johnson KM, Martin DH (1974). Venezuelan equine encephalitis. Adv Vet Sci Comp Med.

[63] Johnstone JA, Ross CA, Dunn M (1972). Meningitis and encephalitis associated with mumps infection. A 10-year survey. Arch Dis Child.

[64] Joki-Korpela P, Marjomaki V, Krogerus C, Heino J, Hyypia T (2001). Entry of human parechovirus 1. J Virol.

[65] Kaiser R (1999). The clinical and epidemiological profile of tick-borne encephalitis in southern Germany 1994-98: a prospective study of 656 patients. Brain.

[66] Kelley TW, Prayson RA, Ruiz AI, Isada CM, Gordon SM (2003). The neuropathology of West Nile virus meningoencephalitis. A report of two cases and review of the literature. Am J Clin Pathol.

[67] Kistler AL, Gancz A, Clubb S, Skewes-Cox P, Fischer K, Sorber K (2008). Recovery of divergent avian bornaviruses from cases of proventricular dilatation disease: identification of a candidate etiologic agent. Virol J.

[68] Klein RS, Lin E, Zhang B, Luster AD, Tollett J, Samuel MA, Engle M, Diamond MS (2005). Neuronal CXCL10 directs CD8+ T-cell recruitment and control of West Nile virus encephalitis. J Virol.

[69] Kopp A, Gillespie TR, Hobelsberger D, Estrada A, Harper JM, Miller RA et al (2013) Provenance and geographic spread of St. Louis encephalitis virus. mBio 4:e00322–13. doi:10.1128/mBio.00322-13 10.1128/mBio.00322-13 PMC3685209 23760463

[70] Korteweg C, Gu J (2008). Pathology, molecular biology, and pathogenesis of avian influenza A (H5N1) infection in humans. Am J Pathol.

[71] Kuiken T, Taubenberger JK (2008). Pathology of human influenza revisited. Vaccine.

[72] Libraty DH, Nisalak A, Endy TP, Suntayakorn S, Vaughn DW, Innis BL (2002). Clinical and immunological risk factors for severe disease in Japanese encephalitis. Trans R Soc Trop Med Hyg.

[73] Lindquist L, Vapalahti O (2008). Tick-borne encephalitis. Lancet.

[74] Love S, Wiley CA, Love S, Louis DN, Ellison DW (2008). Viral diseases. Greenfield’s neuropathology.

[75] Ludlow M (2004) Measles virus: a model for neuronal infection and cell to cell spread. Ph.D. thesis, Queen’s University of Belfast

[76] Ludlow M, Duprex WP, Cosby SL, Allen IV, McQuaid S (2008). Advantages of using recombinant measles viruses expressing a fluorescent reporter gene with vibratome slice technology in experimental measles neuropathogenesis. Neuropathol Appl Neurobiol.

[77] Mansfield KL, Johnson N, Phipps LP, Stephenson JR, Fooks AR, Solomon T (2009). Tick-borne encephalitis virus—a review of an emerging zoonosis. J Gen Virol.

[78] Mardekian SK, Fortuna D, Nix A, Bhatti T, Wiley CA, Flanders A (2015). Severe human parechovirus type 3 myocarditis and encephalitis in an adolescent with hypogammaglobulinemia. Int J Infect Dis.

[79] McJunkin JE, de los Reyes EC, Irazuzta JE, Caceres MJ, Khan RR, Minnich LL LL (2001). La Crosse encephalitis in children. N Engl J Med.

[80] McQuaid S, Cosby SL, Koffi K, Honde M, Kirk J, Lucas SB (1998). Distribution of measles virus in the central nervous system of HIV-seropositive children. Acta Neuropathol.

[81] Meyding-Lamade U, Strank C (2012). Herpesvirus infections of the central nervous system in immunocompromised patients. Ther Adv Neurol Disord.

[82] Miller HG, Stanton JB, Gibbons JL (1957). Acute disseminated encephalomyelitis and related syndromes. Br Med Journal.

[83] Mizuta K, Kuroda M, Kurimura M, Yahata Y, Sekizuka T, Aoki Y (2012). Epidemic myalgia in adults associated with human parechovirus type 3 infection, Yamagata, Japan, 2008. Emerg Infect Dis.

[84] Mohamed M, Mosha F, Mghamba J, Zaki SR, Shieh WJ, Paweska J (2010). Epidemiologic and clinical aspects of a Rift Valley fever outbreak in humans in Tanzania, 2007. Am J Trop Med Hyg.

[85] Morimoto K, Patel M, Corisdeo S, Hooper DC, Fu ZF, Rupprecht CE (1996). dCharacterization of a unique variant of bat rabies virus responsible for newly emerging human cases in North America. Proc Natl Acad Sci USA.

[86] Morrow JI, Dowey KE, Swallow MW (1986). Subacute sclerosing panencephalitis in Northern Ireland: twenty years’ experience. Ulster Med J.

[87] Mrak RE, Young L (1994). Rabies encephalitis in humans: pathology, pathogenesis and pathophysiology. J Neuropathol Exp Neurol.

[88] Muehlenbachs A, Bhatnagar J, Zaki SR (2015). Tissue tropism, pathology and pathogenesis of enterovirus infection. J Pathol.

[89] Nagel MA, Gilden D (2014). Neurological complications of varicella zoster virus reactivation. Curr Opin Neurol.

[90] Nagel MA, Gilden D (2014). Update on varicella zoster virus vasculopathy. Curr Infect Dis Rep.

[91] Nash D, Mostashari F, Fine A, Miller J, O’Leary D, Murray K, Huang A, Rosenberg A, Greenberg A, Sherman M (2001). The outbreak of West Nile virus infection in the New York City area in 1999. N Engl J Med.

[92] Nobach D, Bourg M, Herzog S, Lange-Herbst H, Encarnacao JA, Eickmann M, Herden C (2015). Shedding of infectious borna disease virus-1 in living bicolored white-toothed shrews. PLoS One.

[93] O’Leary DR, Marfin AA, Montgomery SP, Kipp AM, Lehman JA, Biggerstaff BJ, Elko VL, Collins PD, Jones JE, Campbell GL (2004). The epidemic of West Nile virus in the United States, 2002. Vector Borne Zoonotic Dis.

[94] Ooi MH, Lewthwaite P, Lai BF, Mohan A, Clear D, Lim L (2008). The epidemiology, clinical features, and long-term prognosis of Japanese encephalitis in central sarawak, malaysia, 1997–2005. Clin Infect Dis.

[95] Palacios G, Oberste MS (2005). Enteroviruses as agents of emerging infectious diseases. J Neurovirol.

[96] Parboosing R, Bao Y, Shen L, Schaefer CA, Brown AS (2013). Gestational influenza and bipolar disorder in adult offspring. JAMA Psychiatry.

[97] Pekosz A, Phillips J, Pleasure D, Merry D, Gonzalez-Scarano F (1996). Induction of apoptosis by La Crosse virus infection and role of neuronal differentiation and human bcl-2 expression in its prevention. J Virol.

[98] Pellett PE, Roizman B, Knipe DM, Howley PM (2013). Herpesviridae. Field’s virology.

[99] Perry RT, Gacic-Dobo M, Dabbagh A, Mulders MN, Strebel PM, Okwo-Bele JM, Rota PA, Goodson JL, Centers for Disease C, Prevention (2014). Global control and regional elimination of measles, 2000–2012. MMWR Morb Mortal Wkly Rep.

[100] Powers AM, Oberste MS, Brault AC, Rico-Hesse R, Schmura SM, Smith JF (1997). Repeated emergence of epidemic/epizootic Venezuelan equine encephalitis from a single genotype of enzootic subtype ID virus. J Virol.

[101] Racaniello VR (2006). One hundred years of poliovirus pathogenesis. Virology.

[102] Rayamajhi A, Singh R, Prasad R, Khanal B, Singhi S (2006). Clinico-laboratory profile and outcome of Japanese encephalitis in Nepali children. Ann Trop Paediatr.

[103] Reid AH, McCall S, Henry JM, Taubenberger JK (2001). Experimenting on the past: the enigma of von Economo’s encephalitis lethargica. J Neuropathol Exp Neurol.

[104] Reyes MG, Gardner JJ, Poland JD, Monath TP (1981). St Louis encephalitis. Quantitative histologic and immunofluorescent studies. Arch Neurol.

[105] Rhoades RE, Tabor-Godwin JM, Tsueng G, Feuer R (2011). Enterovirus infections of the central nervous system. Virology.

[106] Rodrigues SG, Nunes MR, Casseb SM, Prazeres AS, Rodrigues DS, Silva MO (2010). Molecular epidemiology of Saint Louis encephalitis virus in the Brazilian Amazon: genetic divergence and dispersal. J Gen Virol.

[107] Rotbart HA (2000). Viral meningitis. Semin Neurol.

[108] Russell RR, Donald JC (1958). The neurological complications of mumps. Br Med J.

[109] Ruzek D, Dobler G, Donoso Mantke O (2010). Tick-borne encephalitis: pathogenesis and clinical implications. Travel Med Infect Dis.

[110] Samuel MA, Morrey JD, Diamond MS (2007). Caspase 3-dependent cell death of neurons contributes to the pathogenesis of West Nile virus encephalitis. J Virol.

[111] Santini M, Kutlesa M, Zarkovic K, Drazenovic V, Barsic B (2012). Influenza A 2009 H1N1 encephalitis in adults with viral RNA in cerebrospinal fluid. Scand J Infect Dis.

[112] Schonberger K, Ludwig MS, Wildner M, Weissbrich B (2013). Epidemiology of subacute sclerosing panencephalitis (SSPE) in Germany from 2003 to 2009: a risk estimation. PLoS One.

[113] Sellner J, Hemmer B, Mühlau M (2010). The clinical spectrum and immunobiology of parainfectious neuromyelitis optica (Devic) syndromes. J Autoimmun.

[114] Selvey LA, Wells RM, McCormack JG, Ansford AJ, Murray K, Rogers RJ (1995). Infection of humans and horses by a newly described morbillivirus. Med J Australia.

[115] Shieh WJ, Guarner J, Layton M, Fine A, Miller J, Nash D (2000). The role of pathology in an investigation of an outbreak of West Nile encephalitis in New York, 1999. Emerg Infect Dis.

[116] Shuangshoti S, Thepa N, Phukpattaranont P, Jittmittraphap A, Intarut N, Tepsumethanon V (2013). Reduced viral burden in paralytic compared to furious canine rabies is associated with prominent inflammation at the brainstem level. BMC Vet Res.

[117] Silverman MA, Misasi J, Smole S, Feldman HA, Cohen AB, Santagata S, McManus M, Ahmed AA (2013). Eastern equine encephalitis in children, Massachusetts and New Hampshire, USA, 1970–2010. Emerg Infect Dis.

[118] Simon M, Hernu R, Cour M, Casalegno JS, Lina B, Argaud L (2013). Fatal influenza A(H1N1)pdm09 encephalopathy in immunocompetent man. Emerg Infect Dis.

[119] Smith DR, Bird BH, Lewis B, Johnston SC, McCarthy S, Keeney A (2012). Development of a novel nonhuman primate model for Rift Valley fever. J Virol.

[120] Solomon T, Dung NM, Kneen R, le Thao TT, Gainsborough M, Nisalak A (2002). Seizures and raised intracranial pressure in Vietnamese patients with Japanese encephalitis. Brain.

[121] Solomon T, Fisher AF, Beasley DW, Mandava P, Granwehr BP, Langsjoen H, Travassos Da Rosa AP, Barrett AD, Tesh RB (2003). Natural and nosocomial infection in a patient with West Nile encephalitis and extrapyramidal movement disorders. Clin Infect Dis.

[122] Solomon T, Lewthwaite P, Perera D, Cardosa MJ, McMinn P, Ooi MH (2010). Virology, epidemiology, pathogenesis, and control of enterovirus 71. Lancet Infect Dis.

[123] Solomon T, Vaughn DW (2002). Pathogenesis and clinical features of Japanese encephalitis and West Nile virus infections. Curr Top Microbiol Immunol.

[124] Southern PM, Smith JW, Luby JP, Barnett JA, Sanford JP (1969). Clinical and laboratory features of epidemic St. Louis encephalitis. Ann Intern Med.

[125] Srinivasan A, Burton EC, Kuehnert MJ, Rupprecht C, Sutker WL, Ksiazek TG (2005). Transmission of rabies virus from an organ donor to four transplant recipients. N Engl J Med.

[126] Steiner I, Benninger F (2013). Update on herpes virus infections of the nervous system. Curr Neurol Neurosci Rep.

[127] Studahl M (2003). Influenza virus and CNS manifestations. J Clin Virol.

[128] Taira N, Kamei S, Morita A, Ishihara M, Miki K, Shiota H, Mizutani T (2009). Predictors of a Prolonged Clinical Course in Adult Patients with Herpes Simplex Virus Encephalitis. Intern Med.

[129] Tapparel C, Siegrist F, Petty TJ, Kaiser L (2013). Picornavirus and enterovirus diversity with associated human diseases. Infect Genet Evol.

[130] Udow SJ, Marrie RA, Jackson AC (2013). Clinical features of dog- and bat-acquired rabies in humans. Clin Infect Dis.

[131] van Marle G, Antony J, Ostermann H, Dunham C, Hunt T, Halliday W (2007). West Nile virus-induced neuroinflammation: glial infection and capsid protein-mediated neurovirulence. J Virol.

[132] van Riel D, Leijten LM, Verdijk RM, GeurtsvanKessel C, van der Vries E, van Rossum AM (2014). Evidence for influenza virus CNS invasion along the olfactory route in an immunocompromised infant. J Infect Dis.

[133] van Thiel PP, de Bie RM, Eftimov F, Tepaske R, Zaaijer HL, van Doornum GJ (2009). Fatal human rabies due to Duvenhage virus from a bat in Kenya: failure of treatment with coma-induction, ketamine, and antiviral drugs. PLoS Negl Trop Dis.

[134] van Velden DJ, Meyer JD, Olivier J, Gear JH, McIntosh B (1977). Rift Valley fever affecting humans in South Africa: a clinicopathological study. S Afr Med J.

[135] Vandercam T, Hintzen RQ, de Boer JH, Van der Lelij A (2008). Herpetic encephalitis is a risk factor for acute retinal necrosis. Neurology.

[136] Verboon-Maciolek MA, Groenendaal F, Hahn CD, Hellmann J, van Loon AM, Boivin G, de Vries LS (2008). Human parechovirus causes encephalitis with white matter injury in neonates. Ann Neurol.

[137] Wasay M, Diaz-Arrastia R, Suss RA, Kojan S, Haq A, Burns D, Van Ness P (2000). St Louis encephalitis: a review of 11 cases in a 1995 Dallas, Tex, epidemic. Arch Neurol.

[138] Wassilak SGF, Oberste MS, Tangermann RH, Diop OM, Jafari HS, Armstrong GL (2014). Progress toward global interruption of wild poliovirus transmission, 2010–2013, and tackling the challenges to complete eradication. J Infect Dis.

[139] Weaver SC, Ferro C, Barrera R, Boshell J, Navarro JC (2004). Venezuelan equine encephalitis. Ann Rev Entomol.

[140] Winkler WG, Fashinell TR, Leffingwell L, Howard P, Conomy P (1973). Airborne rabies transmission in a laboratory worker. JAMA.

[141] Winter PM, Dung NM, Loan HT, Kneen R, Wills B, le Thu T (2004). Proinflammatory cytokines and chemokines in humans with Japanese encephalitis. J Infect Dis.

[142] Wohlsein P, Baumgärtner W, Kreipe HH, Haverich A, Hori A, Stan AC (2011). Rabies transmission through organ transplantation. Der Pathologe.

[143] Wolthers KC, Benschop KS, Schinkel J, Molenkamp R, Bergevoet RM, Spijkerman IJ, Kraakman HC, Pajkrt D (2008). Human Parechoviruses as an important viral cause of sepsislike illness and meningitis in young children. Clin Infect Dis.

[144] Wong KT, Munisamy B, Ong KC, Kojima H, Noriyo N, Chua KB, Ong BB, Nagashima K (2008). The distribution of inflammation and virus in human enterovirus 71 encephalomyelitis suggests possible viral spread by neural pathways. J Neuropathol Exp Neurol.

[145] Wong KT, Robertson T, Ong BB, Chong JW, Yaiw KC, Wang LF, Ansford AJ, Tannenberg A (2009). Human Hendra virus infection causes acute and relapsing encephalitis. Neuropathol App Neurobiol.

[146] Wong KT, Shieh WJ, Kumar S, Norain K, Abdullah W, Guarner J (2002). Nipah virus infection: pathology and pathogenesis of an emerging paramyxoviral zoonosis. Am J Pathol.

[147] Wong KT, Tan CT (2012). Clinical and pathological manifestations of human henipavirus infection. Curr Top Microbiol Immunol.

[148] Wurtz R, Paleologos N (2000). La Crosse encephalitis presenting like herpes simplex encephalitis in an immunocompromised adult. Clin Infect Dis.

[149] Xiang Z, Li L, Lei X, Zhou H, Zhou Z, He B, Wang J (2014). Enterovirus 68 3C protease cleaves TRIF to attenuate antiviral responses mediated by Toll-like receptor 3. J Virol.

[150] Zhang SY, Jouanguy E, Ugolini S, Smahi A, Elain G, Romero P (2007). TLR3 deficiency in patients with herpes simplex encephalitis. Science.

