# Supplementary material for: Neurotropic virus infections as the cause of immediate and delayed neuropathology
Source: Acta Neuropathol. 2015 Dec 10;131:159–84. doi: 10.1007/s00401-015-1511-3 (PMC4713712; doi:10.1007/s00401-015-1511-3)
Supplement: Supplementary file 1 — Supplementary material 1 (DOCX 93 kb) [file 401_2015_1511_MOESM1_ESM.docx]

**Neurotropic virus infections as the cause of immediate and delayed neuropathology**

Martin Ludlow^1^, Jeroen Kortekaas^2^, Christiane Herden^3^, Bernd Hoffmann^4^, Dennis Tappe^5,6^, Corinna Trebst^7^, Diane E. Griffin^8^, Hannah E. Brindle^9,10^, Tom Solomon^9,11^, Alan S. Brown^12^, Debby van Riel^13^, Katja C. Wolthers^14^, Dasja Pajkrt^15^, Peter Wohlsein^16^, Byron E.E. Martina^13, 17^, Wolfgang Baumgärtner^16,18^, Georges M. Verjans^13,1^, Albert D.M.E. Osterhaus^1,17,18^*

^1^ Research Center for Emerging Infections and Zoonoses, University of Veterinary Medicine, Hannover D-30559, Germany

^2^ Department of Virology, Central Veterinary Institute, part of Wageningen University and Research Centre, Wageningen, The Netherlands

^3^Institute of Veterinary Pathology, Justus-Liebig-University Gießen, Gießen, Germany

^4^Institute of Diagnostic Virology, Friedrich-Loeffler-Institut, Greifswald-Insel Riems, Germany

^5^Bernhard Nocht Institute for Tropical Medicine, Hamburg, Germany

^6^German Centre for Infection Research (DZIF), Hamburg, Germany

^7^ Department of Neurology, Hannover Medical School, Hannover, Germany

^8^ W. Harry Feinstone Department of Molecular Microbiology and Immunology, Johns Hopkins Bloomberg School of Public Health , Baltimore, Maryland

^9^ Institute of Infection and Global Health, University of Liverpool, Liverpool, UK

^10^ Wellcome Trust Liverpool Glasgow Centre for Global Health Research, University of Liverpool, Liverpool, UK

^11^ NIHR Health Protection Research Unit in Emerging Infection and Zoonoses, Liverpool, UK

^12^ Department of Psychiatry, Columbia University Medical Center, New York State Psychiatric Institute, New York, NY

^13^ Department of Viroscience, Erasmus MC, Rotterdam, The Netherlands

^14^ Laboratory of Clinical Virology, Department of Medical Microbiology, Academic Medical Center, Amsterdam, The Netherlands

^15^ Department of Pediatric Infectious Diseases, Emma Children's Hospital, Academic Medical Center, Amsterdam, The Netherlands

^16^ Department of Pathology, University of Veterinary Medicine, Hannover, Germany

^17^ Artemis One Health, Utrecht, the Netherlands

^18^ Center of Systems Neuroscience, Hannover, Germany

*** Corresponding author:** Albert D.M.E. Osterhaus, Research Center for Emerging Infections and Zoonoses, University of Veterinary Medicine, Bünteweg 17, Hannover D-30559, Germany; email: [albert.osterhaus@tiho-hannover.de](mailto:albert.osterhaus@tiho-hannover.de)

**Diagnosis and therapy of neurotropic virus infections**

**Alphaviruses**

Eastern equine encephalitis virus (EEEV) is difficult to isolate or even detect by polymerase chain reaction (PCR) in clinical samples. Diagnosis of acute or recent EEEV infections is therefore usually established by detection of IgM antibodies in serum or cerebrospinal fluid (CSF) or by a 4-fold increase in IgG levels in paired serum samples. Enzyme-linked immunosorbent assay (ELISA) or hemagglutination-inhibition assays can be used for initial testing, although plaque-reduction neutralization assays should be performed to exclude cross-reactivity with related alphaviruses. Laboratory examination of CSF may reveal pleocytosis with neutrophil predominance and elevated protein levels with normal glucose levels. Computed tomography (CT) and magnetic resonance imaging (MRI) may reveal focal lesions in the basal ganglia thalami, brain stem and cerebral cortex [28,9].

Venezuelan equine encephalitis (VEEV) can be isolated from blood during the first 3-5 days of illness. In a subset of patients, the virus can also be isolated from pharyngeal swabs. Virus isolation procedures should be performed with great care, considering the high infectivity of the virus via aerosols. As for EEEV, VEEV infection can be diagnosed by detection of IgM antibodies or a 4-fold or greater increase of IgG antibodies in paired samples. Serological differentiation between VEEV and related alphaviruses can be performed by plaque-reduction neutralization tests (PRNT). Laboratory abnormalities are common and include elevated hepatic transaminases and lactate dehydrogenase, lymphocytopenia, eosinophilia and thrombocytopenia [5]. Analysis of CSF may reveal mononuclear pleocytosis, moderately elevated protein levels with normal glucose levels. MRI or CT scans may reveal hemorrhages or edema.

Acute Chikungunya virus (CHIKV) infection can be diagnosed by detection of viral RNA during the first week of disease. IgM antibodies can be detected from day 5 after onset of symptoms. In general, analysis of CSF does not reveal major abnormalities, although pleocytosis is sometimes reported. Laboratory findings may include lymphopenia and in some cases thrombocytopenia. MRI of neonates with severe neurological signs may reveal white matter lesions, brain swelling and cerebral hemorrhage. MRI scans of hospitalized children or adults generally do not reveal abnormalities. In rare cases, neuroimaging of adult patients may reveal bilateral frontoparietal white matter lesions with restricted diffusion, which is considered an early sign of viral encephalitis [11]. Pathomorpologically, there are subarachnoidal cerebellar hemorrhages and severe brain edema, also in the white matter. Focal ischemic changes are located in the frontal and occipital cortexes and the internal capsule as well as in the subcortical white matter. There is only mild microglial activation in the cortical gray matter and diencephalon, however, microglial nodule formation, astrocytosis, neuronophagia and viral inclusion do not occur. Mild perivascular lymphocytic infiltrates and gitter cells may be present in the basal ganglia [11].

There are no registered antiviral treatments to control encephalitic alphaviruses. Supportive therapy includes treatment of seizures with fosphenytoin. Careful monitoring of respiration, fluid balance and cardiac rhythm is important as well as prevention of deep vein thrombosis and secondary bacterial infections. When clinically indicated, increased intracranial pressure lowering therapies may be required.

**Bornaviridae**

The diagnosis of Bornavirus infections in humans has a controversial history relating to the specificity of many of the tests for human infections and issues relating to reproducibility of published studies. A good summary of these diagnostic tests has been published previously by Kathryn M. Carbone [6] . In future, metagenomic approaches will most likely have the greatest chance of identifying isolated Bornavirus infections in the human population. There is currently no curative treatment regime available for Bornavirus infections and vaccine strategies are hampered by the underlying immunopathological processes. However, some antiviral strategies, such as the use of ribavirin, have shown promising results in *in vitro* and *in vivo* model systems [15,22,29]. The continuous emergence and identification of new Bornaviruses in various species warrants further research into therapeutic options for treatment of future zoonotic Bornavirus infections.

**Bunyaviridae**

La Crosse virus (LACV) has never been isolated from blood samples. Therefore, laboratory diagnosis of LACV infection depends on the detection of IgM antibodies in serum or CSF or detection of a four-fold or greater rise in IgG antibodies in paired samples, to be demonstrated by ELISA and confirmed by PRNT. Electroencephalography (EEG) often reveals abnormalities, including background slowing and periodic lateralized epileptiform discharges (PLEDS), usually with involvement of the temporal lobe, similar as can result from herpes simplex encephalitis [21,18]. CT scans do not reveal abnormalities in the majority of LACV-infected patients. When abnormalities are detected, generalized cerebral edema is most common. Patients that do not show abnormalities upon admission may show generalized or multifocal edema after cerebral herniation [18]. MRI examinations may reveal edema, abnormalities suggestive of meningoencephalitis or acute demyelinating encephalomyelitis [21,37]. Most hospitalized patients survive LACV infection, albeit sometimes with sustained neurological sequelae, manifesting as school difficulties, attention-deficit-hyperactivity disorder (ADHD) and short-term memory deficits. The antiviral drug ribavirin has been used for treatment of LACV infections, although the results of these limited studies failed to demonstrate a beneficial effect [19]. The potential serious outcome of LACV infection in young children underscores the need for effective and safe antiviral therapies or even a vaccine.

Toscana virus (TOSV) infection may be diagnosed by detection of the virus in clinical samples during the acute stage of the infection. However, considering the short duration of viremia and genetic variation among isolates, detection of anti-TOSV antibodies in serum and CSF is the preferred means of diagnosis. Antibodies can be detected by ELISA, preferably based on recombinant nucleoprotein, since this is least likely to detect antibodies against related phleboviruses. In most patients, CT and MRI scans do not reveal abnormalities. In sporadic severe cases, hydrocephalus, diffuse atrophy or edema may be detected and EEG may show signs of diffuse encephalopathy and/or diffuse or symmetrical slow waves [2]. Considering the generally benign course of TOSV infections, no antiviral treatments are currently being evaluated. Nevertheless, considering the wide distribution and anticipated further spread of the habitat of TOSV, a vaccine to protect people living in high-risk areas as well as tourists would be of value.

Acute or recent infections with Rift Valley fever virus (RVFV) can diagnosed by detection of viral RNA or IgM antibodies in blood by PCR and ELISA, respectively, or by virus isolation. An increase of IgG antibodies can be detected by ELISA or PRNT. Only a single report on detailed neuroimaging has been reported previously [1]. These examinations, together with clinical signs and symptoms, suggested cerebral vasculitis. Due to the absence of registered antivirals, treatment of RVF depends on supportive therapy. Although numerous fatalities and long-term sequelae following central nervous system (CNS) disease were reported, insufficient data is available to solidly determine the general outcome of RVFV-mediated encephalitis. However, the demonstrated potential of RVFV to cause explosive outbreaks with severe disease in humans explains the need for effective antiviral therapies as well as a vaccine, both of which are currently not available.

**Flaviviridae**

Diagnosis is generally based on the presence of Japanese encephalitis virus (JEV) or West Nile virus (WNV) specific IgM in CSF or serum using an ELISA. The diagnosis of St Louis Encephalitis virus (SLEV) is usually made by IgM capture ELISA or immunofluorescence performed on CSF samples. MRI may show edema of the substantia nigra [33]. There are currently no specific antivirals available for treatment of flavivirus encephalitis. Seizures are effectively controlled using antiepileptic’s such as phenytoin or carbamazepine. Adequate hydration and sodium replenishment are also important for survival. Trials with corticosteroid treatment with the aim of reducing raised intra-cranial pressure or treatment of acute flaccid paralysis (AFP) have also showed no significant statistical benefit of using steroids over placebo. Flaviviruses have been shown to be sensitive to IFN-alpha *in vitro*. However, when the benefit of interferon alfa-2a was assessed *in vivo* no significant difference between treated and control groups in terms of survival or length of time of recovery was seen in patients infected with JEV or WNV. Although ribavirin has been shown to be effective *in vitro*, it is not used for treatment of JEV or WNV infection. There is evidence that use of Intravenous immunoglobulin (IVIG) can even result in better immunity against flavivirus infections [26]. Little is known about potential treatment of SLEV infections. Although the use of monoclonal antibodies may be useful in the future, current treatment is based on supportive measures including correction of hyponatremia and treatment of secondary bacterial infections. Prevention against JEV is the main key to control of the disease. There are four classes of vaccine against JEV including inactivated mouse brain-derived vaccines, inactivated Vero cell-derived vaccines, live attenuated vaccines, and live recombinant (chimeric) vaccines. However, no vaccine is commercially available to prevent infection with either WNV or SLEV. Immunoglobulin has been recommended for use as post-exposure prophylaxis for tick-borne encephalitis virus (TBEV). However, due to concerns about antibody-dependent enhancement of disease in children, use of immunoglobulins was abandoned [32]. There is a safe and effective vaccine available for prevention of TBE. Taken into account that TBEV is an arbovirus, individual protection in endemic areas is not dependent on herd immunity. Therefore, the WHO recommends vaccination of everybody living in hyperendemic areas***.***

**Herpesviridae**

Various factors including host genetics, age and immune status determine the clinical presentation and prognosis of human herpesviruses (HHV) infections of the CNS [31]. Early diagnosis and proper treatment are essential for a favourable outcome. In immunosuppressed patients the diagnosis is challenging as the clinical manifestations may be atypical. Diagnostic algorithms should ideally include specific clinical, neuroimaging and laboratory features. Analysis of CSF is the gold standard in demonstrating the aetiology of viral CNS infection. Whereas general CSF analysis (e.g. white blood cell counts, oligoclonal Ig bands and albumin/glucose levels) can help to identify an infectious aetiology, these are not specific for a particular causative agent. Quantification of intrathecal virus-specific nucleic acids (both DNA and RNA) has revolutionized virological diagnostics of HHV-and other viral-induced CNS diseases [16]. However, PCR data should be interpreted with care as negative and positive results do not refute nor prove the causative role of the virus in clinical manifestations concomitant with active infection. False negative PCR results are commonly obtained when CSF is obtained at <3 days or at >2 weeks of onset of disease and/or in patients receiving antiviral treatment [16]. When clinical symptoms indicate HHV infection but initial PCRs are negative, serial CSF studies are advocated to prove HHV infection. Moreover, demonstration of intrathecal HHV-specific IgG production (antigen-specific indices) is of additive and important value at >2 weeks after onset of disease. False positive intrathecal PCR data could be observed in the setting of lymphotropic HHV in immunosuppressed patients [16,31]. Virus detection may represent benign reactivation of latent virus due to concurrent disease stress (e.g. Human cytomegalovirus (CMV) and Epstein-Barr virus (EBV)), atypical intrathecal infiltration of leukocytes with chromosomal-integrated virus (HHV6) or merely latently infected cells (e.g. EBV). Concurrent detection of latent viral transcripts and local IgG levels specific for the respective HHV in CSF is recommended to differentiate atypical and true HHV-induced neurological disease [31].

Therapy of herpes Simplex Virus (HSV) and varicella zoster virus (VZV) associated neurological diseases includes high dose systemic acyclovir (ACV) therapy for at least 2 weeks [20,13]. In case of ACV-refractory disease due to ACV-resistant viruses, incidentally observed in immunosuppressed patients, antivirals with different drug interaction sites including foscarnet (targeting DNA polymerase) and helicase-primase inhibitors (Pritelivir and ASP2151) are optional [8]. In case of CMV and HHV6 neurological disease, antiviral therapy involves ganciclovir, and the nephrotoxic alternatives foscarnet and cidovovir [8]. Letermovir, a new agent targeting the CMV terminase subunit pUL56, has been tested as CMV prophylaxis in transplant recipients with promising results in reducing the incidence of infections and acceptable safety profile [7]. Contrastingly, treatment of neurological EBV infections are more symptom-orientated because virus-specific antiviral therapy is not available, ACV and ganciclovir are commonly provided without clear evidence [8].

**Orthomyxoviridae**

In general, influenza viruses are not included in routine diagnostics of CSF from suspected cases of viral CNS disease without confirmed influenza virus respiratory symptoms. Since influenza viruses can cause severe encephalitis/encephalopathy without initial respiratory disease, this might indicate that there are many undiagnosed cases. Antiviral treatment available for influenza viruses include neuraminidase inhibitors and inhibitors of the viral M2 protein. Due to increasing resistance for M2 inhibitors, neuraminidase inhibitors are currently most commonly used [24]. The effectiveness of neuraminidase inhibitors for influenza virus-associated CNS disease has not been proven but the fact that oseltamivir availability is low within the CNS might indicate that it is not beneficial [14].

**Paramyxoviridae**

Neurological complications resulting from paramyxovirus infections are typically diagnosed based on the clinical presentation of acute cases e.g. acute demyelinating encephalomyelitis (ADME) or aseptic mumps meningitis. Typically classical clinical signs of acute measles virus (MV) or mumps virus (MuV) infection are concomitant or shortly precede neurological complications. Henipavirus infection is often suspected based on the geographical location of the patient (or recent travel history) and exposure to risk factors via food consumption (date palm sap, Nipah virus (NiV)) or occupational hazard (exposure to clinically ill horses, Hendra virus (HeV)). In all cases of paramyxovirus CNS infections, MRI provides invaluable data on the temporal disease course and brain regions involved. In patients who have a paramyxovirus infection with a longer disease course such as subacute sclerosing panencephalitis (SSPE) or measles inclusion body encephalitis (MIBE), diagnosis is based on clinical presentation, RT-PCR detection of viral genome in the CSF and in the case of SSPE/MIBE, high levels of measles specific IgG in the CSF. In some instances, paramyxovirus CNS infections are only confirmed following biopsies or analysis of brain sections post-mortem [10].

No commercially available antivirals have been shown to be effective in alleviating or altering the outcome of paramyxovirus CNS infections. In the case of ADME, supportive care and the reduction of inflammation via steroid treatments is recommended. Only palliative supportive therapy is recommended for SSPE and MIBE patients as no efficacious antivirals or therapies are available; in all cases the disease is invariably fatal. In the absence of effective treatment(s), the most effective preventative measure is the immunity against MV conferred by measles-mumps-rubella (MMR) vaccination. With the exception of isolated examples [3], all viruses sequenced or isolated from SSPE and MIBE brain tissue has been found to be of a wild-type MV strain lineage. The typical aseptic meningitis caused by MuV is mild and usually resolves with supportive care. In the more complicated cases of MuV encephalitis, there are few treatment options available aside from supportive therapies. The most effective method for preventing the CNS complications associated with MuV infection is the immunity conferred by MMR vaccination. Neurological complications are a typical feature of henipavirus infections in humans but in the absence of approved antivirals, only supportive care can currently be offered to patients. However, the recent development of the humanized monoclonal antibody m102.4 and the validation of its efficacy against both HeV and NiV infection in the African green monkey model should result in a more effective antiviral treatment [4,12]. Finally, the recent introduction of a commercial vaccine against HeV for horses in Australia should in the long-term reduce the exposure of farmers and veterinary staff to this virus.

**Picornaviridae**

The diagnosis of CNS infections caused by picornaviruses is based on detection of the causative agent by PCR and typically (but not exclusively) involves analyses of CSF. Elevated leukocyte counts in CSF (pleocytosis) are in the majority of the cases not present. This is in contrast to other infectious CNS infections. Therefore, normal CSF findings certainly do not rule out a meningitis or encephalitis. Enterovirus (EV) or human parechovirus (HPeV) infected patients usually have normal leukocyte blood count and C-reactive protein remains low or slightly elevated. Although the ultimate proof of disease is the detection of EV or HPeV in CSF by specific RT-PCRs, the diagnosis of EV or HPeV CNS disease can also be made by RT-PCR in other clinical materials from a patient with clear neurological symptoms [17]. Various clinical samples such as stool, throat swabs, and to a lesser extent blood can be used for detection of EVs and HPeVs. EV and HPeV are detectable in stool for a prolonged time and are not always associated with clinical disease. Nevertheless, stool samples are required for diagnosing PV, and HPeV3 in stool samples is strongly associated with clinically relevant disease [36], indicating that stool samples should be included when diagnosing EV/HPeVs.

Up to date there is not a single vaccine registered for non-polio EVs, but currently an inactivated EV71 whole-virus vaccine [38], effective in reducing the burden of both EV71 induced hand-foot-mouth disease and CNS disease, is awaiting approval from the Beijing Drug Administation. Treatment options for EV and HPeV CNS disease remain limited. Successful treatment of severe EV in immune-compromised hosts with the broad anti-enteroviral capsid inhibitor pleconaril is incidentally reported [35]. Pleconaril was rejected by the FDA for use against the common cold in 2002 due to possible drug interactions and today pleconaril is no longer available. Successful treatment with therapeutic immunoglobulin therapy (e.g. IVIG) in patients with primary immune deficiencies with an EV meningitis/encephalitis has been reported. Also in neonates, IVIG has shown to reduce the burden of disease in some cases. Differences in treatment outcomes could be explained by variations in neutralizing antibody titers against different EV types [34]. In summary, there is a need for treatment for severe human picornavirus infections, and several promising antivirals have shown *in vitro* or *in vivo* activity against major EV types [30]. The question is not if, but when antipicornaviral therapy will become available in the clinic.

**Rhabdoviridae**

Rabies is one of oldest recognized infectious diseases in humans, with a clinical presentation at late stages in the disease that is often sufficiently typical to establish the clinical diagnosis of rabies. Despite the availability of several techniques developed for laboratory diagnosis, it remains a largely underreported disease worldwide. Techniques to detect infectious virus, rabies antigen, or viral nucleic acid are available each with their intrinsic advantages and limitations. For several decades the method of choice for laboratory diagnosis of rabies has been the propagation of the virus *in vivo* -by inoculating new-born mice with biopsy material from suspected rabies patients- or by virus isolation in cell culture, usually mouse neuroblastoma cells. Both these techniques are laborious, expensive and time consuming, as final results may take up to two to three weeks. In addition, recent data suggest that the sensitivity of virus isolation is low since it largely depends on the quality of sample collection and preservation, and the amount of infectious virus in the specimen. Detection of viral RNA with real-time PCR (RT-PCR) is currently used as an alternative method to virus isolation in many diagnostic labs. Quality of samples is as crucial for the success of this technique as for virus isolation, however, RT-PCR is more sensitive, less laborious and results are available within hours instead of weeks. With the use of specific probes, genotyping of the infecting virus is also possible within a short period of time. RT-PCR is a relatively expensive test requiring specialized equipment and expensive reagents. Direct fluorescent antigen (DFA) detection is probably still the most common diagnostic test used today. Using a fluorescent labelled monoclonal antibody, rabies virus (RABV) antigen can be detected within hours on smear impressions of infected tissues from rabies suspected patients. Although the cold chain is not strictly required for the specimens to be tested by DFA, it lacks sensitivity and requires a fluorescence microscope to read the results.

Recently, a rapid immunohistochemical test (RIT) was developed. Similar to the DFA, rabies antigen is detected but a peroxidase label is used instead of a fluorescent one. This test is as specific and equally sensitive as the DFA test, but requires more simple and thus less expensive equipment. The wider application of the RIT to diagnose rabies, also in laboratories that cannot regularly perform rabies diagnostics with RT-PCR or DFA may have potential for the future. Besides combatting rabies at the animal source and implementing education and awareness campaigns [23], preventive vaccination of people at risk of exposure is the most effective way of combatting human rabies. Although human vaccines are available, the cost associated with preventive vaccination is often prohibitive in resource poor settings [25].

To date treatment options for rabies are limited. Post exposure treatment protocols using vaccines and specific rabies immunoglobulin preparations are considered to be successful if implemented within 48 hours after exposure [27]. Of all reported symptomatic cases of rabies, only a handful has been reported to have survived, albeit with severe sequelae. All treatment strategies that have been tested so far were based on empirical data and ‘medical intuition’ rather than on scientific evidence or indications that these approaches could work. For example, antiviral agents such as IFN-α and ribavirin have been administered to rabies patients via different routes (intrathecal and/or intravenous) with no apparent success. However, it is likely that suboptimal pharmacokinetics and a low partition coefficient of IFN-α in combination with the action of the efflux pump may have contributed to the failure of intrathecal IFN-α treatment of rabies. Probably due to the failure of the immune system to mount an adequate adaptive immune response upon infection with RABV, hyper immune serum (rabies-specific immunoglobulins) and monoclonal antibodies have been used therapeutically aiming to neutralize RABV in the brain. However no clinical efficacy was observed when given after appearance of the first clinical symptoms of rabies. Medically induced coma, initially practised to reduce suffering, has resulted in saving the lives of a handful of rabies patients. Unfortunately the success rate of this form of therapy is apparently extremely low. This so–called “Milwaukee protocol” is applied in many countries, particularly in the United States, for the treatment of symptomatic rabies. It is based on the assumption that rabies takes place without involvement of viral or immune-mediated cytopathic effects and therefore should be reversible, and clearance of RABV would simply require an effective intervention by normal immune mediated processes. The principle of the treatment is palliation with very intensive medical treatment and prolongation of the survival until eventually the immune system clears RABV from the brain. This treatment has several limitations and has a very low, if any, success rate. However, the Milwaukee protocol was used in 2004 on a non-immunised 15-year old girl infected with bat rabies, and remarkably she survived. The supportive coma-induction, anti-excitatory and antiviral therapies aimed to minimise neurological disturbance whilst the host immune response resulted in elimination of the virus. Assessment of the efficacy of the protocol, and in particular the role of antiviral medication, in subsequent attempts has been confounded by the multiple variations in stage of presentation, exposure history and specific treatment regimes.

**References**

1. Alrajhi AA, Al-Semari A, Al-Watban J (2004) Rift Valley fever encephalitis. Emerg Infect Dis 10:554-555. doi: 10.3201/eid1003.020817

2. Baldelli F, Ciufolini MG, Francisci D, Marchi A, Venturi G, Fiorentini C et al (2004) Unusual presentation of life-threatening Toscana virus meningoencephalitis. Clin Infect Dis 38:515-520. doi: 10.1086/381201

3. Bitnun A, Shannon P, Durward A, Rota PA, Bellini WJ, Graham C et al (1999) Measles inclusion-body encephalitis caused by the vaccine strain of measles virus. Clin Infect Dis 29:855-861. doi: 10.1086/520449

4. Bossart KN, Geisbert TW, Feldmann H, Zhu Z, Feldmann F, Geisbert JB et al (2011) A neutralizing human monoclonal antibody protects african green monkeys from hendra virus challenge. Sci Trans Med 3:105ra103. doi: 10.1126/scitranslmed.3002901

5. Bowen GS, Fashinell TR, Dean PB, Gregg MB (1976) Clinical aspects of human Venezuelan equine encephalitis in Texas. Bull Pan Am Health Organ 10:46-57

6. Carbone KM (2001) Borna disease virus and human disease. Clin Microbiol Rev 14:513-527. doi:10.1128/CMR.14.3.513-527.2001

7. Chemaly RF, Ullmann AJ, Stoelben S, Richard MP, Bornhäuser M, Groth C et al (2014) Letermovir for Cytomegalovirus Prophylaxis in Hematopoietic-Cell Transplantation. N Engl J Med 370:1781-1789. doi: 10.1056/nejmoa1309533

8. De Clercq E (2012) Human viral diseases: what is next for antiviral drug discovery? Curr Opin Virol 2:572-579. doi: 10.1016/j.coviro.2012.07.004

9. Deresiewicz RL, Thaler SJ, Hsu L, Zamani AA (1997) Clinical and neuroradiographic manifestations of eastern equine encephalitis. N Engl J Med 336:1867-1874. doi: 10.1056/NEJM199706263362604

10. Eyre TA, Pelosi E, McQuaid S, Richardson D, Newman J, Hill K, Veys P, Davies G, Orchard KH (2013) Mumps virus encephalomyelitis in a 19-year old male patient with an undefined severe combined immunodeficiency post-haematopoietic bone marrow transplantation: a rare fatal complication. J Clin Virol 57:165-168. doi: 10.1016/j.jcv.2013.02.003

11. Ganesan K, Diwan A, Shankar SK, Desai SB, Sainani GS, Katrak SM (2008) Chikungunya encephalomyeloradiculitis: report of 2 cases with neuroimaging and 1 case with autopsy findings. Am J Neuroradiol 29:1636-1637. doi: 10.3174/ajnr.A1133

12. Geisbert TW, Mire CE, Geisbert JB, Chan YP, Agans KN, Feldmann F et al (2014) Therapeutic treatment of Nipah virus infection in nonhuman primates with a neutralizing human monoclonal antibody. Sci Trans Med 6:242ra282. doi: 10.1126/scitranslmed.3008929

13. Gilden DH, Mahalingam R, Cohrs RJ, Tyler KL (2007) Herpesvirus infections of the nervous system. Nat Clin Pract Neurol 3:82-94. doi: 10.1038/ncpneuro0401

14. Hoffmann G, Funk C, Fowler S, Otteneder MB, Breidenbach A, Rayner CR, Chu T, Prinssen EP (2009) Nonclinical pharmacokinetics of oseltamivir and oseltamivir carboxylate in the central nervous system. Antimicrob Agents Chemother 53:4753-4761. doi: 10.1128/AAC.01541-08

15. Jordan I, Briese T, Averett DR, Lipkin WI (1999) Inhibition of Borna disease virus replication by ribavirin. J Virol 73:7903-7906

16. Kleines M, Scheithauer S, Schiefer J, Häusler M (2014) Clinical application of viral cerebrospinal fluid PCR testing for diagnosis of central nervous system disorders: a retrospective 11-year experience. Diagn Microbiol Infect Dis 80:207-215. doi: 10.1016/j.diagmicrobio.2014.07.010

17. Manukyan M, Triantafilou K, Triantafilou M, Mackie A, Nilsen N, Espevik T, Wiesmuller KH, Ulmer AJ, Heine H (2005) Binding of lipopeptide to CD14 induces physical proximity of CD14, TLR2 and TLR1. Eur J Immunol 35:911-921. doi: 10.1002/eji.200425336

18. McJunkin JE, de los Reyes EC, Irazuzta JE, Caceres MJ, Khan RR, Minnich LL, Fu KD, Lovett GD, Tsai T, Thompson A (2001) La Crosse encephalitis in children. N Engl J Med 344:801-807. doi: 10.1056/NEJM200103153441103

19. McJunkin JE, Nahata MC, De Los Reyes EC, Hunt WG, Caceres M, Khan RR et al (2011) Safety and pharmacokinetics of ribavirin for the treatment of la crosse encephalitis. Pediatr Infect Dis J 30: 860-865. doi: 10.1097/INF.0b013e31821c922c

20. Meyding-Lamade U, Strank C (2012) Herpesvirus infections of the central nervous system in immunocompromised patients. Ther Adv Neurol Disord 5:279-296. doi: 10.1177/1756285612456234

21. Miller A, Carchman R, Long R, Denslow SA (2012) La Crosse viral infection in hospitalized pediatric patients in Western North Carolina. Hospital Pediatr 2:235-242

22. Mizutani T, Inagaki H, Araki K, Kariwa H, Arikawa J, Takashima I (1998) Inhibition of Borna disease virus replication by ribavirin in persistently infected cells. Arch Virol 143:2039-2044

23. Muller T, Freuling CM, Wysocki P, Roumiantzeff M, Freney J, Mettenleiter TC, Vos A (2015) Terrestrial rabies control in the European Union: historical achievements and challenges ahead. Vet J 203:10-17. doi: 10.1016/j.tvjl.2014.10.026

24. Nguyen-Van-Tam JS, Venkatesan S, Muthuri SG, Myles PR (2015) Neuraminidase inhibitors: who, when, where? Clin Microbiol Infect 21:222-225. doi: 10.1016/j.cmi.2014.11.020

25. Publication WHO (2010) Rabies vaccines: WHO position paper--recommendations. Vaccine 28:7140-7142. doi: 10.1016/j.vaccine.2010.08.082

26. Rayamajhi A, Nightingale S, Bhatta NK, Singh R, Ledger E, Bista KP et al (2015) A preliminary randomized double blind placebo-controlled trial of intravenous immunoglobulin for Japanese encephalitis in Nepal. PloS One 10: e0122608. doi: 10.1371/journal.pone.0122608

27. Salve H, Kumar S, Sa R, Rai SK, Kant S, Pandav CS (2014) Feasibility of sustainable provision of intradermal post exposure prophylaxis against rabies at primary care level--evidence from rural Haryana. BMC Health Serv Res 14:278. doi: 10.1186/1472-6963-14-278

28. Silverman MA, Misasi J, Smole S, Feldman HA, Cohen AB, Santagata S, McManus M, Ahmed AA (2013) Eastern equine encephalitis in children, Massachusetts and New Hampshire,USA, 1970-2010. Emerg Infect Dis 19:194-201.. doi: 10.3201/eid1902.120039

29. Solbrig MV, Schlaberg R, Briese T, Horscroft N, Lipkin WI (2002) Neuroprotection and reduced proliferation of microglia in ribavirin-treated bornavirus-infected rats. Antimicrob Agents Chemother 46:2287-2291

30. Thibaut HJ, Leyssen P, Puerstinger G, Muigg A, Neyts J, De Palma AM (2011) Towards the design of combination therapy for the treatment of enterovirus infections. Antiviral Res 90:213-217. doi: 10.1016/j.antiviral.2011.03.187

31. Venkatesan A, Tunkel AR, Bloch KC, Lauring AS, Sejvar J, Bitnun A et al (2013) Case Definitions, Diagnostic Algorithms, and Priorities in Encephalitis: Consensus Statement of the International Encephalitis Consortium. Clin Infect Dis 57:1114-1128. doi: 10.1093/cid/cit458

32. Waldvogel K, Bossart W, Huisman T, Boltshauser E, Nadal D (1996) Severe tick-borne encephalitis following passive immunization. Eur J Pediatr 155:775-779

33. Wasay M, Diaz-Arrastia R, Suss RA, Kojan S, Haq A, Burns D, Van Ness P (2000) St Louis encephalitis: a review of 11 cases in a 1995 Dallas, Tex, epidemic. Arch Neurol 57:114-118

34. Wildenbeest JG, Harvala H, Pajkrt D, Wolthers KC (2010) The need for treatment against human parechoviruses: how, why and when? Expert Rev Anti Infect Ther 8:1417-1429. doi: 10.1586/eri.10.130

35. Wildenbeest JG, van den Broek PJ, Benschop KSM, Koen G, Wierenga PC, Vossen ACTM, Kuijpers TW, Wolthers KC (2011) Pleconaril revisited: clinical course of chronic enteroviral meningoencephalitis after treatment correlates with in vitro susceptibility. Antivir Ther 17:459-466. doi: 10.3851/imp1936

36. Wildenbeest JG, Wildenbeest JG, Benschop KSM, Minnaar RP, Bouma-de Jongh S, Wolthers KC, Pajkrt D, Benschop KSM, Minnaar RP, Bouma-de Jongh S, Wolthers KC, Pajkrt D (2014) Clinical relevance of positive human parechovirus type 1 and 3 PCR in stool samples. Clin Microbiol Infect 20:O640-O647. doi: 10.1111/1469-0691.12542

37. Wurtz R, Paleologos N (2000) La Crosse encephalitis presenting like herpes simplex encephalitis in an immunocompromised adult. Clin Infect Dis 31:1113-1114. doi: 10.1086/321804

38. Zhu F, Xu W, Xia J, Liang Z, Liu Y, Zhang X et al (2014) Efficacy, Safety, and Immunogenicity of an Enterovirus 71 Vaccine in China. N Engl J Med 370:818-828. doi: 10.1056/nejmoa1304923
